# The effect of oat β-glucan on postprandial blood glucose and insulin responses: a systematic review and meta-analysis

**DOI:** 10.1038/s41430-021-00875-9

**Published:** 2021-02-19

**Authors:** Andreea Zurbau, Jarvis C. Noronha, Tauseef A. Khan, John L. Sievenpiper, Thomas M. S. Wolever

**Affiliations:** 1 INQUIS Clinical Research Ltd. (formerly GI Labs), Toronto, ON Canada; 2 Toronto 3D Knowledge Synthesis and Clinical Trials Unit, Clinical Nutrition and Risk Factor Modification Centre, St. Michael’s Hospital, Toronto, ON Canada; 3 Department of Nutritional Sciences, Temerty Faculty of Medicine, University of Toronto, Toronto, ON Canada; 4 Department of Medicine, Temerty Faculty of Medicine, University of Toronto, Toronto, ON Canada; 5 Division of Endocrinology and Metabolism, Department of Medicine, St. Michael’s Hospital, Toronto, ON Canada; 6 Li Ka Shing Knowledge Institute, St. Michael’s Hospital, Toronto, ON Canada

**Keywords:** Nutrition therapy, Diabetes

## Abstract

To determine the effect of oat β‑glucan (OBG) on acute glucose and insulin responses and identify significant effect modifiers we searched the MEDLINE, EMBASE, and Cochrane databases through October 27, 2020 for acute, crossover, controlled feeding trials investigating the effect of adding OBG (concentrate or oat-bran) to carbohydrate-containing test-meals compared to comparable or different carbohydrate-matched control-meals in humans regardless of health status. The primary outcome was glucose incremental area-under-the-curve (iAUC). Secondary outcomes were insulin iAUC, and glucose and insulin incremental peak-rise (iPeak). Two reviewers extracted the data and assessed risk-of-bias and certainty-of-evidence (GRADE). Data were pooled using generic inverse-variance with random-effects model and expressed as ratio-of-means with [95% CIs]. We included 103 trial comparisons (*N* = 538). OBG reduced glucose iAUC and iPeak by 23% (0.77 [0.74, 0.81]) and 28% (0.72 [0.64, 0.76]) and insulin by 22% (0.78 [0.72, 0.85]) and 24% (0.76 [0.65, 0.88]), respectively. Dose, molecular-weight, and comparator were significant effect modifiers of glucose iAUC and iPeak. Significant linear dose-response relationships were observed for all outcomes. OBG molecular-weight >300 kg/mol significantly reduced glucose iAUC and iPeak, whereas molecular-weight <300 kg/mol did not. Reductions in glucose iAUC (27 vs 20%, *p* = 0.03) and iPeak (39 vs 25%, *p* < 0.01) were significantly larger with different vs comparable control-meals. Outcomes were similar in participants with and without diabetes. All outcomes had high certainty-of-evidence. In conclusion, current evidence indicates that adding OBG to carbohydrate-containing meals reduces glycaemic and insulinaemic responses. However, the magnitude of glucose reduction depends on OBG dose, molecular-weight, and the comparator.

## Introduction

β-glucan, a viscous soluble dietary fibre found naturally in oats and barley, has a number of potentially beneficial physiological effects which include reducing both postprandial glycaemic responses (PPGR) [1] and serum cholesterol [2–4]. The ability of β-glucan to reduce PPGR was established by a European Food Safety Authority (EFSA) Panel review that concluded that 4 g of either oat β-glucan (OBG) or barley β-glucan (BBG) per 30 g of available carbohydrates (avCHO) is required to obtain a consistent reduction in PPGR [5]. A subsequent review confirmed this by showing that OBG and BBG significantly reduced PPGR in all studies that used doses >4 g/30 g avCHO [1]. However, we found that adding 1.7 g or 2.5 g OBG/30 g avCHO to instant oats or muffin significantly reduced glucose iAUC by 16% [6] and 24% [7], respectively. Thus, the amount of OBG required to obtain a clinically meaningful reduction in PPGR is not clear. The estimate of 4 g OBG/30 g avCHO is imprecise because the previous reviews did not address the confounding effects of β-glucan source and molecular weight (MW) and their results are not expressed in such a way that clinical relevance can be assessed.

Both EFSA [5] and Tosh [1] included studies using oats or barley grains, flakes or flour as sources of β-glucan. This is a problem because each gram of β-glucan in oats and barley, respectively, is accompanied by ~13 g and ~11 g avCHO whose effect on glucose and insulin responses is influenced to a large extent by cooking and processing [8, 9]. Thus, the inclusion of whole grains, flakes and flour confounds the effect of β-glucan in potentially unpredictable ways. Furthermore, since BBG differs from OBG with respect to the ratio of β-(1 → 3) to β-(1 → 4) linkages, MW, solubility and conformation [10], the effects of purified OBG on PPGR may differ from those of purified BBG. This is supported by the studies cited by Tosh [1] that used purified β-glucan or β-glucan concentrates (OBG, *n* = 41; BBG, *n* = 10). Although all β-glucan doses >4 g/30 g avCHO (OBG, *n* = 18 BBG, *n* = 1) significantly reduced PPGR, smaller doses reduced PPGR more often with OBG than BBG, 15 of 23 (65%) vs 2 of 7 (22%, *χ*^2^ *p* = 0.028). Therefore, we excluded studies using BBG from this analysis.

The MW and dose of OBG have independent effects on PPGR [7]. EFSA [5] did not consider MW, and by excluding treatments where the β-glucan MW had been deliberately reduced to <250,000 g/mol, Tosh [1] was unable to assess the effect of MW on PPGR.

Tosh [1] found that 4 g β-glucan/30 g avCHO reduced glucose iAUC by an average of 27 ± 3 mmol×min/L relative to a variety of different comparators. However, the clinical relevance of this difference depends on the population studied and the nature of the comparator. For example, blood glucose iAUC in 77 subjects without diabetes varied from ~80 to ~550 mmol × min/L after consuming 50 g glucose, and ~40 to ~450 mmol × min/L after 50 g avCHO from white bread [11]. Thus, a 27 mmol × min/L difference in iAUC is equivalent to reductions varying from 5–34% relative to glucose or 6–68% relative to white bread. Assessing the relative differences within trials and considering the nature of the comparator test-meal may provide more precise and clinically meaningful estimates of the effect of OBG on PPGR.

For these reasons, we aimed to synthesize the evidence from acute, crossover, single-meal, controlled feeding trials of the effect of OBG on postprandial glucose and insulin responses in humans regardless of health status, and to explore whether OBG dose, MW, nature of the comparator, health status, OBG food form, study methodology quality, duration of follow-up and risk of bias modified these effects.

## Methods

This systematic review and meta-analysis was conducted according to the Cochrane Handbook for Systematic Reviews of Interventions [12]. Data were reported in accordance with the Preferred Reporting Items for Systematic Reviews and Meta-Analyses (PRISMA) guidelines [13]. The study protocol was registered on the Open Science Forum (OSF) registry [14].

### Data sources

MEDLINE, EMBASE, and the Cochrane Central Register of Controlled Trials were searched through October 27, 2020. Electronic searches were supplemented with manual searches of references from included studies. The detailed search strategy is outlined in Supplementary Table 1.

### Study selection

We included randomized and non-randomized acute, crossover, single-meal, controlled feeding trials that investigated the effects of OBG or oat bran (high in OBG) added to a carbohydrate-containing meal in humans regardless of health status on at least one of the following 4 variables: glucose and insulin incremental area-under-the-curve (iAUC) and incremental peak-rise (iPeak). To be included, the comparator (control) test-meal had to contain an equivalent amount of avCHO as the OBG-containing test-meal; however, the sources of avCHO did not have to be the same. In some studies, the sources of avCHO in the control and the OBG test-meals were comparable (matched control; e.g., OBG-containing spaghetti vs OBG-free spaghetti) and in some they were different (unmatched control; e.g., OBG-containing spaghetti vs white bread). We excluded trials of parallel design, chronic feeding, studies in which participants were not fasting at baseline, and trials that did not provide appropriate outcome data, used non-oat sources of β-glucan (e.g., BBG), and used oats or oat flour as the sole source of OBG. We excluded non-oat sources of β-glucan because, as explained above, their structure and effect on glycaemic responses differs from that of OBG. We excluded studies using oats or oat flour as the sole source of OBG because each gram of OBG in oats or oat flour is accompanied by about 13 g avCHO which itself influences glucose and insulin responses in ways which are affected by cooking and processing [8] and, thus, confounds the effect of OBG in potentially unpredictable ways.

### Data extraction

Two investigators (AZ and JCN) independently reviewed and extracted relevant data from each included report. Extracted data included participant characteristics (e.g., health status, age, sex, BMI), OBG dose (expressed as g OBG/30 g avCHO [5]), OBG MW (< 300 kg/mol = low; 300 to <1000 kg/mol = medium; ≥1000 kg/mol = high), intervention and comparator meal characteristics (nature of foods [e.g. glucose, bread, muffins, pasta, juice] and macronutrient composition [total carbohydrate, total fibre, soluble fibre, available carbohydrate (avCHO), protein, fat]), study design, duration of follow-up, setting, funding sources, and outcome data. In the absence of numerical values for outcome data and the inability to contact study authors, values were extracted from figures using Plot Digitizer, version 2.5.1 (Free Software Foundation, Boston, MA). If outcome data were provided for multiple follow-up durations (e.g., 120 min and 180 min) in a single study, the data points for the 120 mins were preferred to minimize heterogeneity. Similarly, matched control comparisons were preferred to unmatched control comparisons when available. The same two investigators also assessed risk of bias and study methodology quality from each included report. Risk of bias was evaluated using version 2 of the Cochrane risk-of-bias (RoB 2) tool, where bias was assessed in five distinct domains (bias arising from the randomization process, bias due to deviations from intended interventions, bias due to missing outcome data, bias in measurement of the outcome, and bias in selection of the reported result). Within each domain, the investigators answered one or more signalling questions and these answers led to judgements of “low risk of bias”, “some concerns”, or “high risk of bias” [10, 15]. Study methodology quality was assessed for each trial comparison based on eleven predetermined criteria outlined in Supplementary Table 2 which was adapted from a previous study [11, 16]. This assessment identified protocol components that have been established as significant determinants of the accuracy and precision of PPGR measurements [17–28]. The score was used to inform the responses to the signalling questions in the “bias in measurement of the outcome” domain of the RoB 2 tool and to determine the influence of study methodology quality in the relationship between OBG and PPGR. One of the criteria (criteria #10) was specific to iAUC methodology and was excluded for iPeak outcomes. Studies were categorized as having high methodology quality and a low risk of bias in measurement of the outcome if they specified the OBG dose and met ≥7 or ≥6 of the other criteria for iAUC and iPeak outcomes, respectively. Studies which did not meet these thresholds were considered to have low methodology quality and some concerns for risk of bias in the measurement of the outcome. Any discrepancies in data extraction, risk of bias, and study methodology quality assessments were reconciled by consensus with a third reviewer (TMSW).

### Outcomes

The primary outcome was glucose iAUC. Secondary outcomes were insulin iAUC, glucose iPeak and insulin iPeak.

### Data synthesis and analysis

The pooled effect estimate for each outcome was expressed as a ratio of means (RoM) with 95% confidence intervals (CIs). RoM is a method to present continuous measures on a ratio scale and is calculated by dividing the mean value in the intervention group by the mean value in the control group (Supplementary Fig. 1). This method facilitates clinical interpretation (e.g., RoM of 1.2 indicates an increase of 20% in the intervention group compared to the control group; a RoM of 0.7 indicates a reduction of 30%) and controls for baseline differences in the comparator groups across studies [29–31]. Paired analyses were applied to all comparisons [31, 32]. If multiple comparisons were available in the same population, we controlled for unit of analysis error by dividing the N of the respective arm by the number of times it was included.

STATA Version 16 (StataCorp, TX, USA) was used to conduct all analyses. Natural log-transformed RoM (ln[RoM]) and standard error (SE) of the ln[RoM] were pooled using the generic inverse variance method with DerSimonian and Laird random effects models [33, 34]. Fixed effects models were only used if fewer than 5 trials were present for an outcome [35]. Linear dose-response was modelled using one-stage random effects with restricted maximum likelihood methods assuming a linear function [36]. Non-linear dose response was modelled with restricted cubic splines with three knots. The Wald’s test was used to examine departure from linearity.

Inter-study heterogeneity was assessed using the Cochran Q statistic and quantified using the I^2^ statistic, where *I*^2^ ≥ 50% and *P*_Q_ < 0.10 were considered evidence of substantial heterogeneity [12]. Potential sources of heterogeneity were investigated by sensitivity analyses and subgroup analyses. For determination of whether a single trial comparison exerted undue influence, sensitivity analyses were performed in which we recalculated the pooled effect estimates and heterogeneity after removing each individual trial. Studies whose removal explained the heterogeneity, changed the direction or significance of the effect, or altered the effect size by 10% or more were considered influential. We conducted a priori subgroup and publication bias analyses for all relationships with ≥10 trial comparisons. A priori and post hoc categorical subgroup analyses were conducted using meta-regression with a *P* < 0.05 indicative of a significant difference between subgroups. A priori subgroups included health status, OBG dose, comparator, food form, duration of follow-up, risk of bias, and study methodology quality. By mistake, MW was not included in the registered list of subgroups, and, since this was not discovered until after the initial analysis had been completed, we cannot prove that examination of the effect of MW was done a priori; therefore it has to be considered as a *post hoc* subgroup analysis. Publication bias was assessed by visual inspection of funnel plots for asymmetry and formal testing by the Egger’s and Begg’s tests [37, 38]. If evidence of publication bias was detected, Duval and Tweedie nonparametric “trim and fill” analyses were applied to assess the effect of the imputed “missing” studies [39].

### Grading of the evidence

The overall certainty of the evidence was evaluated using the GRADE tool where the certainty of the evidence was graded as high, moderate, low, or very low certainty [40–42]. Randomized controlled trials are graded as high certainty evidence by default and then downgraded on the basis of the following pre-specified criteria: risk of bias (weight of studies shows important risk of bias as assessed by the RoB2 tool), inconsistency (substantial unexplained inter-study heterogeneity, *I*^2^ ≥ 50% and *P*_Q_ < 0.10), indirectness (presence of factors that limit generalizability of the results), imprecision, and publication bias (significant evidence of small-study effects). For the purposes of GRADE we considered the results to be imprecise if the 95% CIs of the pooled effect estimates were wide or overlapped 0.8 (equivalent to a 20% reduction in glucose iAUC which Health Canada considered to be the minimum physiologically relevant difference [43]). Certainty of the evidence was upgraded if a dose-response was detected.

## Results

### Search results

We identified a total of 1522 reports, of which 1353 were excluded based on title and/or abstract review (Supplementary Fig. 2). The remaining 169 articles were reviewed in full and 135 were excluded. A total of 35 reports [6, 7, 44–76], containing data for 103 trial comparisons involving 538 participants, met the eligibility criteria and were included in the final analyses.

### Trial characteristics

Table 1 and Supplementary Table 3 show the summary and individual characteristics of all included trials and trial comparisons. The median follow-up duration across all trials was 120 min (range 60–240). All trials were in outpatient settings with the majority conducted in North America (68%) and Europe (24%). Trial funding came from agency sources (42%), industry sources (27%), or both (9%), with no funding information reported in 22% of the trials. Participants were males and females (50% male, 50% female) aged (median (range) of the reported means) 37 (22–66) years with a BMI of 24.9 (20.6–31.1) kg/m². Most trials were conducted in healthy participants (75%) with some in individuals with type 2 diabetes (11%), those who were overweight (9%), both healthy and overweight (5%), or had metabolic syndrome (1%).

**Table 1:** Summary of trial characteristics.

Characteristic^a^	
Trial comparisons	103
Participants	538
Follow-up duration, minutes	120 (60–240)
Participant characteristics	
• Age, years	37 (22–66)
• Male:female^b^ (%)	50:50
• BMI, kg/m^2^	24.9 (20.6–31.1)
Health Status, # of trial comparisons	
• Healthy	77
• Type 2 Diabetes	11
• Overweight	9
• Mixed (Health & Overweight)	5
• Metabolic Syndrome	1
Intervention characteristics	
• OBG dose, g	4.2 (0.2–11.7)
• OBG dose, g/30 g available carbohydrates	2.8 (0.1–22.6)
• Food source, # of trials	
⚬ OBG-enriched/oat bran muffins	23
⚬ OBG/oat bran added to glucose/dextrose	20
⚬ OBG-enriched/oat bran cereal	13
⚬ OBG-enriched/oat bran added to oatmeal porridge	13
⚬ Oat granola/muesli with oat bran flakes	10
⚬ OBG-enriched bread	8
⚬ OBG-enriched/oat bran snack bar or product	7
⚬ OBG-enriched/oat bran beverage (juice, shake, drink)	7
⚬ Oat bran pasta	2
• Available carbohydrate, g	50 (13–75)
Comparator characteristics	
• Type	
⚬ Matched	66
⚬ Unmatched	37
• Food source, # of trial comparisons	
⚬ Glucose/dextrose/maltodextrin solution	32
⚬ White bread	21
⚬ Muffin	16
⚬ Wheat porridge or oatmeal without added OBG	12
⚬ Wheat granola/muesli with cornflakes	7
⚬ Snack bar/product	5
⚬ Cornflakes cereal	4
⚬ Juice/shake/drink	4
⚬ Durum wheat pasta	2
• Available carbohydrate, g	50 (13–75)
Setting, # of trial comparisons	
• North America	70
• Europe	25
• South America	4
• Australia	3
• Asia	1
Funding source, # of trial comparisons^c^	
• Agency	43
• Industry	28
• Agency & Industry	9
• Not reported	23

*OBG* oat β-glucan, *BMI* body mass index.

^a^Median (range) of mean data, unless otherwise indicated.

^b^30/31 studies provided data on sex.

^c^Agency funding is that from government, university, or not-for-profit sources. Industry funding is that from trade organizations that obtain revenue from the sale of products.

The interventions involved consumption of OBG added to various foods including muffins (22%), glucose/dextrose solutions and gels (19%), cereals (13%), porridges (13%), muesli (10%), bread (8%), snack bars or products (7%), juices/shakes/drinks (7%) and pasta (2%). Four food form categories were identified: gels, liquids, semi-solids (hot cereals) and solids (bread, muffins, food bars and granola). A median (range) of 4.2 (0.2–11.7) g OBG was added to a median 50 (13–75) g avCHO, for a median OBG dose per 30 g avCHO of 2.8 (0.1–22.6) g. The comparators in these trials included glucose/dextrose/maltodextrin solutions (31%), white bread (20%), muffins (16%), porridge or oatmeal (12%), granola or muesli with cornflakes (7%), snack bars (5%), cornflakes cereal (4%), juice/shakes/drinks (4%), and pasta (2%) without added OBG.

### Risk of bias

Supplementary Tables 4–7 show individual risk of bias assessments. Low study methodology quality was identified in 23%, 41%, 3% and 15% of trial comparisons for iAUC glucose and insulin and iPeak glucose and insulin, respectively, and were assessed as having some concerns for risk of bias in the measurement of the outcome. Supplementary Figs. 3–6 show summary risk of bias assessments. Most trials included for glucose iAUC and iPeak were assessed as having low overall risk of bias whereas, 71% of trials for insulin iAUC either had some concerns or high overall risk of bias and 69% of trials for iPeak insulin had some concerns for overall risk of bias.

### Effect of oat β-glucan on glucose iAUC

**Fig. 1:**
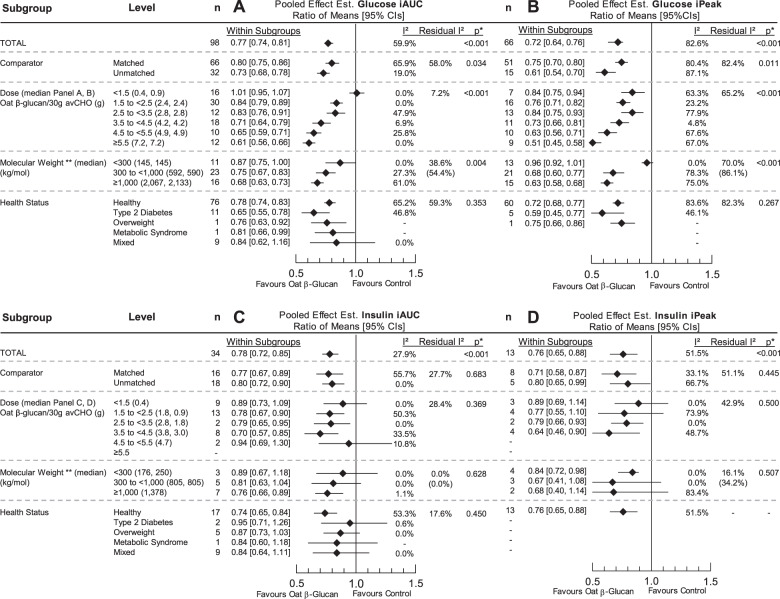
Effect of oat β-glucan on postprandial glycaemic and insulinaemic responses. Pooled effect estimates of the effect of oat β-glucan on the incremental area under the curve (iAUC) for blood glucose (**A**), incremental peak rise (iPeak) for blood glucose (**B**), iAUC insulin (**C**), and iPeak insulin (**D**). Pooled effect estimates are expressed as ratios of means (RoMs, black diamond) with 95% CIs (solid lines). Pooled analyses were conducted using the generic inverse variance method with random effects models. Interstudy heterogeneity was tested by the Cochran Q statistic (χ^2^) at a significance level of *P*_Q_ < 0.10 (not shown) and quantified by I^2^. The residual I^2^ value represents unexplained heterogeneity for each subgroup. n, number of trial comparisons. *Differences between subgroups were tested using meta-regression and the significance level was reported as a *p* value, where *p* < 0.05 was considered significant.

In 98 trial comparisons involving 508 participants, pooled analysis showed that OBG reduced glucose iAUC by 23% (RoM 0.77 [95% CI 0.74, 0.81], *p* < 0.001 with substantial heterogeneity, *I*^2^ = 59.9%, *P*_Q_ < 0.001) (Fig. 1A). Removal of individual trials did not alter the direction, significance, or magnitude (10% or more) of the effect or the evidence for heterogeneity. There was significant effect modification by comparator (*p* = 0.034, residual *I*^2^ = 58.0%, *P*_Q_ < 0.001), OBG dose (*p* < 0.001, residual *I*^2^ = 7.2%, *P*_Q_ = 0.284), and OBG MW (*p* = 0.004, residual *I*^2^ = 38.6%, *P*_Q_ = 0.004), (Fig. 1A, Supplementary Figs. 7–9). Only OBG doses >1.5 g/30 g avCHO and MW > 300 kg/mol were shown to significantly reduce glucose iAUC. Duration was a significant effect modifier (*p* = 0.021, residual *I*^2^ = 58.3%, *P*_Q_ < 0.001) with the largest reduction seen for a postprandial period of 240 min which was limited to one study with 3 trial comparisons (Supplementary Fig. 10A). Food form was also a significant effect modifier (*p* = 0.045, residual *I*^2^ = 60.1%, *P*_Q_ < 0.001) with the effect of liquid food forms being significantly less than solid (*p* = 0.01) and semi-solid (*p* = 0.02) (Supplementary Fig. 10A).

No significant effect modifications were observed by health status (*p* = 0.35, residual *I*^2^ = 59%, *P*_Q_ < 0.001), study methodology quality (*p* = 0.226, residual *I*^2^ = 59.9%, *P*_Q_ < 0.001) and overall risk of bias (*p* = 0.61, residual *I*^2^ = 60%) (Fig. 1A, Supplementary Figs. 10A, 11–12).

**Fig. 2:**
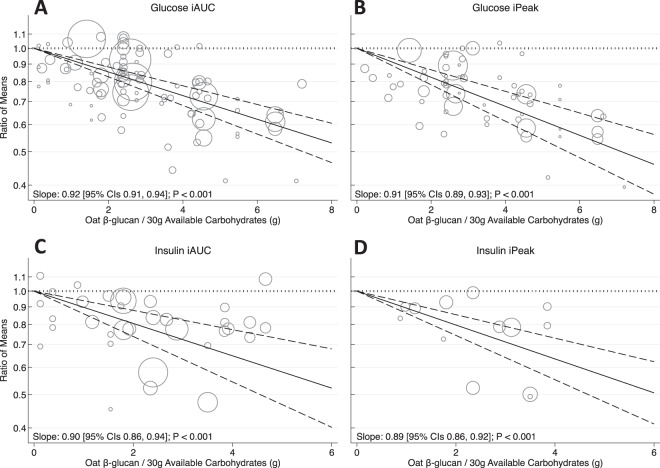
Effect of oat β-glucan dose on postprandial glycaemic and insulinaemic responses. Pooled dose-response relationship between oat β-glucan and incremental area under the curve (iAUC) for blood glucose (**A**), incremental peak rise (iPeak) for blood glucose (**B**), iAUC insulin (**C**), and iPeak insulin (**D**). Changes in the outcomes (y-axis) are presented as ratios of means (RoMs). Oat β-glucan dose is presented on a 1 g/30 g available carbohydrate scale. Individual comparisons are represented by the circles, with the weight of the study in the overall analysis represented by the size of the circles. The central straight line represents the fitted dose response estimate with outer dashed lines representing the 95% confidence intervals (CIs), which was modelled using one-stage random effects with the generic inverse variance and restricted maximum likelihood methods, assuming a linear function.

**Fig. 3:**
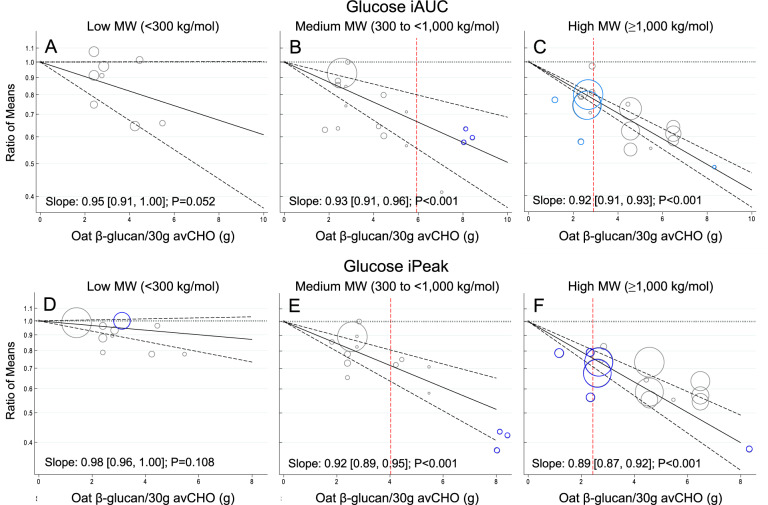
Effect of oat β-glucan molecular weight and dose on postprandial glycaemic responses. Pooled dose-response relationship by low, medium and high molecular weight oat β-glucan on the incremental area under the curve (iAUC) for blood glucose (**A**–**C**) and incremental peak rise (iPeak) for blood glucose (**D**–**F**). Changes in the outcomes (y-axis) are presented as ratios of means (RoMs). Oat β-glucan dose is presented on a 1 g/30 g available carbohydrate scale. Individual comparisons are represented by the circles, with the weight of the study in the overall analysis represented by the size of the circles. The light grey and blue circles represent the trial comparisons with matched and unmatched comparators, respectively. The central straight line represents the fitted dose response estimate with outer dashed lines representing the 95% confidence intervals (CIs), which was modelled using one-stage random effects with the generic inverse variance and restricted maximum likelihood methods, assuming a linear function. The vertical dashed line represents the dose level at which the upper-bound 95% CIs cross the physiologically relevant threshold of a 20% reduction.

A significant linear dose-response relationship was observed suggesting an 8% reduction in glucose iAUC per 1 g OBG/30 g avCHO (slope 0.92 [95% CI 0.91, 0.94], *p* < 0.001) (Fig. 2A and Supplementary Fig. 13). When this relationship was assessed based on MW (Fig. 3A–C), medium and high MW OBG demonstrated significant dose-response relationships (*p* < 0.001) whereas, low MW OBG did not (*p* = 0.052). The dose-response with high MW OBG was more precise than with medium MW OBG (slope 0.92 [0.91, 0.93] vs. 0.93 [0.91, 0.96], respectively). Supplementary Figs. 14 and 15 show the dose-response relationship by health status and study methodology quality, respectively. More precise dose-response relationships were observed in trials assessing healthy individuals (slope 0.93 [0.92, 0.94]) compared to those with type 2 diabetes (slope 0.91 [0.86, 0.97]), and in trials of high study methodology quality (slope 0.93 [0.91, 0.94]) compared to those with low study methodology quality (slope 0.91 [0.86, 0.96]). The slope of the dose-response relationship was greater but less precise for the unmatched comparators (0.90 [0.86, 0.93]) than the matched comparators (0.94 [0.92, 0.96]) but the confidence intervals overlapped (Supplementary Fig. 16).

### Effect of Oat β-glucan on glucose iPeak

In 66 trial comparisons involving 313 participants, pooled analysis showed that OBG reduced glucose iPeak by 28% (0.72 [0.64, 0.76], *p* < 0.001, with substantial heterogeneity, *I*^2^ = 82.6%, *P*_Q_ < 0.01) (Fig. 1B). Removal of individual trials did not alter the direction, significance, or magnitude (10% or more) of the effect or the evidence for heterogeneity. There was significant effect modification by the comparator (*p* < 0.011, residual *I*^2^ = 82.4%, *P*_Q_ < 0.001), OBG dose (*p* < 0.001, residual *I*^2^ = 65.2%, *P*_Q_ < 0.001) and OBG MW (*p* < 0.001, residual I^2^ = 70.0%, *P*_Q_ < 0.001) (Fig. 1B, Supplementary Figs. 17–19). Greater reductions in glucose iPeak were observed when OBG containing foods were compared to unmatched comparators than with matched comparators. OBG MW > 300 kg/mol led to greater reductions in glucose iPeak compared to MW < 300 kg/mol. Duration was a significant effect modifier (*p* = 0.024, residual *I*^2^ = 82.8%, *P*_Q_ < 0.001) with the largest reduction seen for a postprandial period of 240 min which was limited to one study with 3 trial comparisons (Supplementary Fig. 10B). Food form was also a significant effect modifier (*p* = 0.029, residual *I*^2^ = 82.8%, *P*_Q_ < 0.001) with a larger reduction observed in meals where OBG was consumed in semi-solid versus liquid form (Supplementary Fig. 10B). No significant effect or heterogeneity modifications were observed by health status (*p* = 0.267, residual *I*^2^ = 82.3%, *P*_Q_ < 0.001), study methodology quality (*p* = 0.347, residual I^2^ = 82.8%, P_Q_ < 0.001), and overall risk of bias (*p* = 0.15, residual *I*^2^ = 82%) (Fig. 1B, Supplementary Figs. 10B, 20–21).

A significant linear dose-response relationship was observed suggesting a 9% reduction in glucose iPeak per 1 g OBG/30 g avCHO (slope 0.91 [95% CI 0.89, 0.93], *p* < 0.001) (Fig. 2B and Supplementary Fig. 13). When this relationship was assessed based on MW (Fig. 3D–F), medium and high MW OBG demonstrated significant dose-response relationships (*p* < 0.001) whereas, low MW OBG did not (*p* = 0.108). Supplementary Figs. 14–16 show the dose-response relationship by health status, study methodology quality, and the comparator. More precise dose-response relationships were observed in trials assessing healthy individuals (slope 0.91 [0.89, 0.94]) compared to those with type 2 diabetes (slope 0.90 [0.83, 0.97]), in high study methodology quality trials (slope 0.91 [0.89, 0.93]) compared to low study methodology quality trials (slope 0.86 [0.76, 0.98]), and matched comparators (slope 0.92 [0.90, 0.94]) compared to unmatched comparators (slope 0.89 [0.85, 0.93]).

### Effect of Oat β-glucan on insulin iAUC

In 34 trial comparisons involving 231 participants, pooled analysis showed that OBG reduced insulin iAUC by 22% (0.78 [0.72, 0.85], *p* < 0.001, with no evidence of heterogeneity, *I*^2^ = 27.9%, *P*_Q_ = 0.10) (Fig. 1C). Removal of individual trials did not alter the direction, significance, or magnitude (10% or more) of the effect or the evidence for heterogeneity. No significant effect modifications were observed by dose, MW, comparator, health status, food form, duration, study methodology quality, and risk of bias (Fig. 1C, Supplementary Figs. 10C, 22–26).

A significant linear dose-response relationship was observed suggesting a 10% reduction in insulin iAUC per 1 g OBG/30 g avCHO (slope 0.90 [95% CI 0.86, 0.94], *p* < 0.001) (Fig. 2C and Supplementary Fig. 13). Health status (not illustrated due to limited data in type 2 diabetes population) and the comparator did not modify the dose-response relationship (Supplementary Fig. 16). A larger dose-response relationship was observed in trials of low study methodology quality (slope 0.82 [0.77, 0.87]) compared to trials of high study methodology quality (slope 0.92 [0.88, 0.96]) (Supplementary Fig. 15).

### Effect of Oat β-glucan on insulin iPeak

In 13 trial comparisons involving 115 participants, pooled analysis showed that OBG reduced insulin iPeak by 24% (0.76 [0.65, 0.88], *p* < 0.001, with no evidence of heterogeneity, *I*^2^ = 51.5%, *P*_Q_ = 0.11) (Fig. 1D). Removal of individual trials did not alter the direction, significance, or magnitude (10% or more) of the effect or the evidence for heterogeneity. No significant effect modifications were observed by dose, MW, comparator, food form, duration, study methodology quality, and risk of bias (Supplementary Figs. 10D, 27–30). Subgroup analysis by health status was not conducted as data were limited to only healthy individuals (Supplementary Fig. 31).

A significant linear dose-response relationship was observed suggesting a 11% reduction in insulin iAUC per 1 g OBG/30 g avCHO (slope 0.89 [95% CI 0.86, 0.92], *p* < 0.001) (Fig. 2D and Supplementary Fig. 13). A significant dose-response relationship was observed in trials of high methodology quality (slope 0.89 [0.86, 0.93]) whereas trials of low study methodology quality did not demonstrate a significant dose-response relationship (slope 0.95 [0.82, 1.10]) (Supplementary Fig. 15). The dose-response relationship was similar when assessed by the comparator (Supplementary Fig. 16).

### Publication bias analyses

Visual inspection of funnel plots for publication bias showed no evidence of asymmetry or small-study effects for glucose and insulin iAUC and iPeak (Supplementary Fig. 32). Both Egger and Begg tests were non-significant for all outcomes.

### GRADE assessment

A summary of the GRADE assessments for each outcome is shown in Supplementary Table 8. Our certainty in the evidence was high for the effect of OBG on reducing glucose and insulin iAUC and iPeak. We identified serious imprecision in the insulin iAUC and iPeak pooled effect estimates, as the upper bounds of the 95% Cis overlapped the minimally important difference of 0.8, which could not be explained by our subgroup analyses. There was evidence of imprecision of the glucose iAUC pooled effect estimate, however subgroup analyses illustrated precise pooled effect estimates at doses ≥3.5 g/30 g available carbohydrates of OBG and from high MW OBG and thus, no downgrade was applied. We also identified serious inconsistency in the glucose iPeak due to significant unexplained heterogeneity (*I*^2^ = 82.6%). Substantial heterogeneity of the pooled effect estimate was observed in glucose iAUC (*I*^2^ = 59.9%), however, the between-study variance was largely explained by dose (residual *I*^2^ = 7.2%, *P*_Q_ = 0.284) and partially by molecular weight (residual *I*^2^ = 38.6%, *P*_*Q*_ = 0.004). Although 71% of trials for insulin iAUC either had some concerns or high overall risk of bias, and 69% of trials for iPeak insulin had some concerns for overall risk of bias, subgroup analyses suggest that risk of bias was not a significant effect modifier of the pooled effect estimates and thus, no downgrades were applied. We upgraded our certainty in the evidence for glucose and insulin iAUC and iPeak due to the presence of dose-response relationships.

## Discussion

### Summary of findings

This systematic review and meta-analysis of acute, crossover, single-meal, controlled feeding trials, including 103 comparisons in 538 participants from 35 reports, showed that OBG reduced glucose and insulin iAUC by 23% and 22%, respectively, and glucose and insulin iPeak by 28% and 24%, respectively. OBG dose, MW, the comparator, intervention food form, and study duration were significant effect modifiers of the reduction in glucose iAUC and iPeak.

Significant linear dose-responses were found for all endpoints, with each g OBG/30 g avCHO reducing glucose iAUC by (mean [95% CI]) 8 [6, 9]%, glucose iPeak by 9 [7, 11]%, insulin iAUC by 10 [6, 14]% and insulin iPeak by 11 [8, 14]%. However, the dose-response relationships for glycaemic response became steeper and had smaller 95% CI as OBG MW increased; for OBG with MW < 300 kg/mol, 300 to <1000 kg/mol and ≥1000 kg/mol, respectively, each g OBG/30 g avCHO reduced glucose iAUC by 5 [9,0]%, 7 [4, 9]% and 8 [7, 9]% and glucose iPeak by 2 [4,0]%, 8 [5, 11]% and 11 [8, 13]%. All outcomes were similar in participants with and without diabetes.

### Results in relation to previous studies

Our findings are consistent with an earlier review by Tosh [1] which showed that OBG elicited a dose-dependent reduction in glucose iAUC and also significantly reduced glucose iPeak and insulin iAUC. However, Tosh expressed the differences in iAUC as absolute values (mmol×min/L), excluded treatments where the β-glucan MW had been deliberately reduced to <250 kg/mol and excluded studies in individuals with diabetes. Our results also show that OBG significantly reduced glycaemic and insulinaemic responses, but expressing the results as RoM rather than absolute values allows the magnitude and clinical utility of the effects to be determined, and allows for valid comparison of the effects in subjects with and without diabetes. Furthermore, by including in our analysis studies using low MW OBG we were able to determine how much low MW OBG reduces glucose and insulin responses in comparison to the effects of medium and high MW OBG.

In substantiating the health claim for OBG and BBG on the reduction of PPGR, the EFSA panel [5] provided a narrative review of 6 studies, 2 of which we included [54, 57] and 4 of which we did not because the sources of β-glucan were oat flour or barley [77–80]. The basis for the panel’s opinion that “…the studies above show an effect of oat and barley beta-glucans in decreasing post-prandial glycaemic responses … at doses of at least 4 g per 30 g available carbohydrates” is difficult to appreciate because information about the number of comparisons, the dose of β-glucan and endpoint used (e.g., iPeak, iAUC) is not provided. Our pooled result from 98 comparisons is similar to the EFSA conclusion in suggesting that a dose of >3.5 g OBG/30 g avCHO is required to obtain a ≥20% reduction in glucose iAUC. However, our results suggest that if high MW OBG is used, ≥20% reductions in glucose iAUC and iPeak can be obtained with 2.9 and 2.5 g OBG /30 g avCHO, respectively. Furthermore, based on 5 studies in participants with diabetes considered by the EFSA panel (only 3 of which we included [47, 56, 69] since the other 2 were not acute test meal studies [81, 82]), the EFSA panel noted that “the evidence provided does not establish that results obtained in patient populations treated with anti-diabetic medications can be generalized to the target population with respect to postprandial glycaemic responses” [4]. The rationale for this conclusion is not clear, but our results show that OBG reduces glycaemic responses to a similar, or perhaps even greater, extent in individuals with type 2 diabetes than in those without diabetes, respectively, for glucose iAUC (35% vs. 22%) and glucose iPeak (41% vs. 28%). This is consistent with the finding that the relative glycaemic responses of foods compared to glucose (i.e., their glycaemic index values) are similar in subjects with and without diabetes [83].

### Effect modifiers and other sources of heterogeneity

Effect modifiers of the effect of OBG on glucose response were OBG dose, MW, the comparator, intervention food form, and study duration, but there were no significant effect modifiers for insulin response.

#### OBG dose and MW

The mechanism by which viscous fibres reduce postprandial glucose and insulin responses is thought to be related to their ability to increase the viscosity of the contents of the gastrointestinal tract (GIT) [84]. The viscosity of OBG solutions is determined by the concentration and MW of the OBG [85]. This is consistent with our finding that both dose and MW significantly modify the effect of OBG on glycaemic response. However, in order to increase the viscosity of the contents of the GIT, the OBG consumed has to be released from the food matrix. The solubility, or bioavailability, of OBG in foods varies [7, 65, 66] and reducing OBG solubility reduces its effect on glycaemic responses [60]. Thus, unmeasured variation in OBG solubility may contribute to the unaccounted for heterogeneity of our results. Furthermore, the viscosity of glucose solutions containing OBG is not always related to their glycaemic impact [59] because the concentration, and hence viscosity, of OBG solutions within the stomach may be reduced by gastric fluid secretions [86]. High viscosity could reduce glycaemic responses by delaying gastric emptying or reducing the rate of digestion and absorption of carbohydrates in the small intestine, or both; however, the exact mechanism is not completely understood. For example, we recently showed that consuming 4 g OBG in a breakfast test-meal reduced both the rate of gastric emptying and the glycaemic response, effects which were abrogated by reducing the dose of OBG or by reducing its MW [76]. Nevertheless, there was no correlation between gastric emptying and glycaemic response elicited by the 4 test meals within the 28 subjects.

#### Comparator

The effect of OBG on glucose responses was greater for unmatched vs matched Control test-meals. The nature of the comparator is important because source of avCHO, quantified by glycaemic-index (the extent to which the avCHO in a food raises glucose iAUC relative to an equal weight of glucose), is an independent determinant of glucose iAUC [87]. For test-meals containing equivalent amounts of avCHO, protein and fat, differences in glycaemic-index are proportional to differences in glucose iAUC [88]. In 26 (81%) of the 32 unmatched comparisons the source of avCHO in the Control test-meals was glucose, dextrose, maltodextrin or white bread, with the source in the other 6 being cream of rice, wheat muffin or cornflakes. If the glycaemic-index of the avCHO in an OBG test-meal is less than that of its Control, the difference in glucose iAUC would be larger than if their glycaemic-indices had been equivalent. The estimated mean glycaemic-index of the unmatched Control test-meals, 81, was 14% greater than that of the OBG test-meals, 71, a difference which could account for the 10% lower mean ROM for the unmatched vs matched comparators 0.73 vs 0.80.

#### Intervention food form

OBG in liquid form had a smaller effect on glycaemic response than in semi-solid or solid food forms. It seems unlikely this is due to food form per se, since, if anything, an equivalent amount of OBG in liquid form, vs solid form, may be more soluble in the gut and, hence, have a greater effect on glycaemic response. The effect modification due to food form is more likely accounted for by the lower MW of the OBG contained in the liquid vs the semi-solid and solid forms. If an OBG-enriched liquid is to remain a palatable liquid, the OBG must be hydrolyzed to reduce its MW and viscosity, whereas high MW OBG can be incorporated into palatable semi-solid and solid foods. This is likely why none of the liquid test meals contained high-MW OBG. The percentage of low-, medium- and high-MW OBG contained in the *n* = 11 liquid forms (45%, 55%, 0%, respectively) differed significantly from that in 11 semi-solid (9%, 55%, 36%, *p* = 0.036) and the 26 solid forms (19%, 35%, 46%, *p* = 0.020), with the distribution in semi-solid and solid forms being similar. An estimate of the effect of MW in the different forms can be obtained by multiplying the mean % reduction for low-, medium- and high-MW OBG (13%, 25% and 32%, respectively, for glucose iAUC) by the respective proportion of low-, medium- and high-MW OBG within each form and summing the products (e,g., the expected iAUC reduction for liquids = 0.13×0.45 + 0.25×0.55 + 0.32 × 0 = 0.20). The expected reductions for glucose iAUC, 20%, 26% and 26% for liquid, semi-solid and solid, respectively, are similar to those observed, 14%, 22%, 25%, as are those for glucose iPeak, 19%, 31%, 29%, vs 22%, 37%, 22%.

#### Study duration

Although study duration was an effect modifier, sensitivity analyses showed that the difference was attributed to the one study with a 4 h postprandial duration. It is known that the method used to calculate iAUC, the duration of blood sampling and the interval between blood samples can influence glucose iAUC [17, 89] and iPeak [20]. Furthermore, the method used by authors to calculate iAUC is often not indicated, and the likelihood of incorrect calculation may be >50% [26]. These factors may contribute towards unexplained heterogeneity in the results.

### Clinical implications

In synthesizing the available data on the effect of OBG on acute glycaemic responses, the question arises as to whether reducing acute glucose and insulin responses has any clinical relevance. Reducing the glycaemic impact of high carbohydrate meals with treatments which reduce the rate of carbohydrate absorption, such as α-glucosidase inhibitors [90, 91] and low glycaemic index foods [92] have desirable physiological effects for many people, particularly for those with pre-diabetes or diabetes. Our findings suggest that the acute effects of OBG on glucose and insulin responses in subjects without diabetes can be extrapolated to people with diabetes. Whether the acute effect of OBG on postprandial glucose response translates into clinically meaningful benefits in long-term glycaemic control is not clear. An earlier systematic review and meta-analysis of 4 randomized controlled trials in 350 individuals with type 2 diabetes found that OBG consumption of 2.5–3.5 g/day significantly lowered HbA_1c_ by 0.21% and fasting plasma glucose by 0.52 mmol/L, without affecting fasting plasma insulin concentrations [93]. However, an updated analysis showed no effect on HbA1c (mean difference, −0.55% [95% CI −1.21, 0.11]) and fasting glucose (−0.54 mmol/L [−1.70, 0.62]) when data were pooled from 5 trials in 535 individuals with type 2 diabetes [94]. Furthermore, the mechanism by which OBG improved glycaemic control in these studies may not be related to an effect on acute glycaemic response but rather to an ability of OBG to favourably alter the colonic microbiome [95]. There is a need for more long-term randomized controlled trials to confirm the effect of OBG on glycaemic control in diabetes and determine a mechanism of action.

If the acute effect of OBG on postprandial glucose responses is beneficial, how large of an effect is required for physiological relevance? Health Canada opined that the minimum physiologically relevant difference in glucose iAUC is 20% [43]. We used this conservative value to assess whether there was imprecision of the results as an indicator of certainty of the evidence. However, differences in diet glycaemic index of 10–15% may be clinically relevant. For example, in a randomized clinical trial of 210 subjects with type 2 diabetes with baseline HbA1c of 7.1% studied for 6 months, a 14% reduction in diet GI was associated with a clinically meaningful reduction of HbA1c of 0.32% relative to control (*p* < 0.001) [96, 97]. Our results suggest that only ~2 g/30 g avCHO of high MW OBG is required to reduce glucose iAUC by 14% with 95% certainty (Fig. 3C).

### Limitations

The pooled effect estimates were imprecise for insulin iAUC and iPeak, as the 95% CIs overlap the minimally important difference for clinical benefit. This imprecision may be due to the limited range of doses included. Although the dose response for each outcome showed that the 95% CIs of the regressions entered the bounds of the minimally important difference as the dose increased, the categorical dose response analysis illustrated imprecision across all dose ranges. Therefore, more studies, including interventions with doses >4.5 g/30 g available carbohydrate of OBG, may improve the precision of the effect of OBG on insulin outcomes. There was also substantial heterogeneity in the overall pooled effect estimate for glucose iPeak, that could only be partially explained by ranges in dose and molecular weight of the included trials.

Of note, our health status category was based on population demographics described in the included studies, therefore it is possible that there may be some overlap between the healthy and overweight categories, in which populations that were categorized as healthy likely also included overweight individuals. Data were also limited in individuals with type 2 diabetes for insulin iAUC and not available for insulin iPeak and thus, more studies would be useful to improve the precision of our findings in this population.

## Conclusion

Our synthesis of the available evidence from acute, crossover, single-meal, controlled feeding trials demonstrates that OBG leads to a clinically meaningful reduction in postprandial glucose responses provided that a sufficient amount of high MW OBG is provided. OBG interventions also resulted in clinically meaningful but imprecise reductions in insulin iAUC and iPeak, for which dose may modify the magnitude of the effect. Health status, OBG food form, postprandial duration, study methodology quality and risk of bias did not meaningfully modify these effects. More studies are needed to improve precision in the effects of OBG in diabetes and to explore whether the acute reductions in glycaemic response translate into clinically meaningful benefits in long-term glycaemic control. These conclusions apply to the addition of purified OBG or oat products highly enriched in OBG to food. Although OBG is present in oats, it is unclear if our findings can be extrapolated to commercial foods containing oats as a source of OBG due to the presence of other nutrients (avCHO, protein and fat) and differences in food processing which may modify these effects.

## Supplementary information

Supplemental Material
