# Supplementary material for: The effect of oat β-glucan on postprandial blood glucose and insulin responses: a systematic review and meta-analysis
Source: Eur J Clin Nutr. 2021 Feb 19;75(11):1540–54. doi: 10.1038/s41430-021-00875-9 (PMC8563417; doi:10.1038/s41430-021-00875-9)
Supplement: Supplementary file 1 — Supplemental Material [file 41430_2021_875_MOESM1_ESM.docx]

**TABLE OF CONTENTS**

[**Abbreviations** 3](#_Toc58934859)

[**Supplementary Table 1.** Search strategy 3](#_Toc58934860)

[**Supplementary Table 2.** Study quality criteria 4](#_Toc58934861)

[**Supplementary Figure 1.** Formulas to calculate ratio of means (RoM) and the standard error of the RoM. 4](#_Toc58934862)

[**Supplementary Figure 2.** Summary of evidence search and selection process 5](#_Toc58934863)

[**Supplementary Table 3.** Study Characteristics 6](#_Toc58934864)

[**Supplementary Table 4.** RoB of trial comparisons on the effect of OBG on glucose iAUC. Trial comparisons were assessed as having some concerns in the measurement of the outcome if either the dose of OBG was not specified or the sum of the other study methodology criteria was <7 10](#_Toc58934865)

[**Supplementary Table 5.** RoB of trial comparisons on the effect of OBG on glucose iPeak. Trial comparisons were assessed as having some concerns in the measurement of the outcome if either the dose of OBG was not specified or the sum of the other study methodology criteria was <6 12](#_Toc58934866)

[**Supplementary Table 6.** RoB of trial comparisons on the effect of OBG on insulin iAUC. Trial comparisons were assessed as having some concerns in the measurement of the outcome if either the dose of OBG was not specified or the sum of the other study methodology criteria was <7 14](#_Toc58934867)

[**Supplementary Table 7.** RoB of trial comparisons on the effect of OBG on insulin iPeak. Trial comparisons were assessed as having some concerns in the measurement of the outcome if either the dose of OBG was not specified or the sum of the other study methodology criteria was <6 15](#_Toc58934868)

[**Supplementary Figure 3.** RoB summary on the effect of OBG on glucose iAUC. . 16](#_Toc58934869)

[**Supplementary Figure 4.** RoB summary on the effect of OBG on glucose iPeak. 16](#_Toc58934870)

[**Supplementary Figure 5**. RoB summary on the effect of OBG on insulin iAUC. 17](#_Toc58934871)

[**Supplementary Figure 6.** RoB summary on the effect of OBG on insulin iPeak. 17](#_Toc58934872)

[**Supplementary Figure 7**. Pooled effect estimates of OBG and glucose iAUC by type of comparator. 18](#_Toc58934873)

[**Supplementary Figure 8.** Pooled effect estimates of OBG dose and glucose iAUC. 20](#_Toc58934874)

[**Supplementary Figure 9.** Pooled effect estimates of OBG molecular weight and glucose iAUC. 23](#_Toc58934875)

[**Supplementary Figure 10**. Summary plot of pooled effect estimates of OBG on glucose and insulin iAUC and iPeak by study postprandial duration, intervention food form and study methodology quality. 25](#_Toc58934876)

[**Supplementary Figure 11.** Pooled effect estimates of OBG and glucose iAUC by health status. 26](#_Toc58934877)

[**Supplementary Figure 12**. RoB subgroup analysis of OBG on glucose iAUC 28](#_Toc58934878)

[**Supplementary Figure 13.** Pooled dose-response relationship (linear and non-linear) between OBG and glucose and insulin iAUC and iPeak. 29](#_Toc58934879)

[**Supplementary Figure 14.** Pooled linear dose-response relationship by health status between OBG and glucose iAUC and iPeak. 30](#_Toc58934880)

[**Supplementary Figure 15.** Pooled linear dose-response relationship by study methodology quality between OBG and glucose and insulin iAUC and iPeak. 31](#_Toc58934881)

[**Supplementary Figure 16.** Pooled linear dose-response relationship by comparator type between OBG and glucose and insulin iAUC and iPeak. 32](#_Toc58934882)

[**Supplementary Figure 17.** Pooled effect estimates of OBG and glucose iPeak by type of comparator. 33](#_Toc58934883)

[**Supplementary Figure 18.** Pooled effect estimates of OBG dose and glucose iPeak. 35](#_Toc58934884)

[**Supplementary Figure 19.** Pooled effect estimates of OBG molecular weight and glucose iPeak. 38](#_Toc58934885)

[**Supplementary Figure 20.** Pooled effect estimates of OBG and glucose iPeak by health status. 40](#_Toc58934886)

[**Supplementary Figure 21**. RoB subgroup analysis of OBG on glucose iPeak 42](#_Toc58934887)

[**Supplementary Figure 22.** Pooled effect estimates of OBG dose and insulin iAUC. 43](#_Toc58934888)

[**Supplementary Figure 23.** Pooled effect estimates of OBG molecular weight and insulin iAUC. 45](#_Toc58934889)

[**Supplementary Figure 24.** Pooled effect estimates of OBG and insulin iAUC by type of comparator. 46](#_Toc58934890)

[**Supplementary Figure 26**. RoB subgroup analysis of OBG on insulin iAUC 49](#_Toc58934891)

[**Supplementary Figure 27.** Pooled effect estimates of OBG dose and insulin iPeak. 50](#_Toc58934892)

[**Supplementary Figure 28.** Pooled effect estimates of OBG molecular weight and insulin iPeak. 51](#_Toc58934893)

[**Supplementary Figure 29.** Pooled effect estimates of OBG and insulin iPeak by type of comparator. 52](#_Toc58934894)

[**Supplementary Figure 30**. RoB subgroup analysis of OBG on insulin iPeak 53](#_Toc58934895)

[**Supplementary Figure 31.** Pooled effect estimates of OBG and insulin iPeak by health status. 54](#_Toc58934896)

[**Supplementary Figure 32.** Contour-enhanced funnel plot for the effect of OBG on the glucose and insulin iAUC and iPeak. 55](#_Toc58934897)

[**Supplementary Table 8.** GRADE certainty of the evidence assessment 56](#_Toc58934898)

# **Abbreviations**

**iAUC** – incremental area under the curve

**iPeak** – incremental peak rise

**OBG –** Oat β-glucan

**PRISMA** – Preferred reporting items for systematic review and meta-analysis protocols

**RoB –** Risk of bias

**RoM** – Ratio of means

# **Supplementary Table 1.** Search strategy

| **MEDLINE**  **1946 to Oct 2, 2019** | **EMBASE**  **1946 to October 2, 2019** | **Cochrane Central Register of Controlled Trials**  **Through Oct 2, 2019** |
| --- | --- | --- |
| 1. oat.tw. | 1. oat.tw. | 1. Avena.ti,ab,kw. |
| 2. Oats.tw. | 2. Oats.tw. | 2. Oat.ti,ab,kw. |
| 3. Avena/ | 3. Avena/ | 3. Oats.ti,ab,kw. |
| 4. Avena*.tw. | 4. Avena*.tw. | 4. Beta-glucans/ |
| 5. beta glucan/ | 5. beta glucan/ | 5. Beta glucan*.ti,ab,kw. |
| 6. beta glucan*.mp. | 6. beta glucan*.mp. | 6. or/1-5 |
| 7. b-blucan*.mp. | 7. b-blucan*.mp. | 7. Glucose/ |
| 8. b-blucan*.mp. | 8. b-blucan*.mp. | 8. glycaemic.mp. |
| 9. or/1-8 | 9. or/1-8 | 9. glycemic.mp. |
| 10. exp Glucose/ | 10. exp Glucose/ | 10. glycemia.mp. |
| 11. glycaemic.mp. | 11. glycaemic.mp. | 11. Insulin/ |
| 12. glycemic.mp. | 12. glycemic.mp. | 12. exp Glucose Tolerance Test/ |
| 13. glycaemia.mp. | 13. glycaemia.mp. | 13. OGTT.mp. |
| 14. glycemia.mp. | 14. glycemia.mp. | 14. or/7-13 |
| 15. exp Glucose Tolerance Test/ | 15. exp Glucose Tolerance Test/ | 15. 6 and 14 |
| 16. OGTT.mp. | 16. OGTT.mp. |  |
| 17. or/10-16 | 17. or/10-16 |  |
| 18. 9 and 17 | 18. 9 and 17 |  |
| 19. limit 18 to animals | 19. limit 18 to animals |  |
| 20. 18 not 19 | 20. 18 not 19 |  |
|  |  |  |

# **Supplementary Table 2.** Study quality criteria

| # | Criteria |
| --- | --- |
| 1 | Was the amount of oat β-glucan/oat bran specified? |
| 2 | Were intervention and comparator meals consumed in different orders? |
| 3 | Were the macronutrient compositions of the intervention and comparator meals specified? |
| 4 | Was the quantity of available carbohydrate in intervention and comparator meals specified? |
| 5 | Was the molecular weight of oat β-glucan specified? |
| 6 | Were intervention and comparator meals consumed with a beverage of >200ml? |
| 7 | Was follow-up duration >120mins? |
| 8 | Was blood collected >7times? |
| 9 | Were duplicate fasting samples collected? |
| 10* | Was the methodology to calculate incremental area under the curve (iAUC) described? |
| 11 | Were participants provided with >3 of the following study visit preparation instructions?   - Fasting for 10-14h - No unusual food intake the day before study visit - No unusual physical activity the day before study visit - No alcohol intake the day before study visit - Provided standard dinner or advised participants to consume usual dinner prior to study visit |

*only applied to iAUC glucose and insulin

**Supplementary Figure 1.** Formulas to calculate ratio of means (RoM) and the standard error of the RoM.

$$\ln\left( RoM \right)=ln(\frac{mean [intervention]}{mean [control]} )$$

$$SE \left[ \ln\left( RoM \right) \right]=\sqrt{\frac{{SD}^{2} \left[ intervention \right]}{n \left[ pairs \right] \times{mean}^{2} \left[ intervention \right]}+\frac{{SD}^{2} \left[ control \right]}{n \left[ pairs \right] \times{mean}^{2}\left[ control \right]}-\frac{2 \times r \times SD \left[ intervention \right] \times SD \left[ control \right]}{mean \left[ intervention \right] \times mean \left[ control \right] \times n [pairs]}}$$

The natural logarithm transformed ratios were aggregated across studies using the standard generalized inverse variance method. The pooled transformed ratio and pooled SE of the logarithmic-transformed ratio were then back transformed to obtain a pooled ratio and 95% CI [29-31] “r” refers to the correlation between the two measurements and was assumed to be 0.5. “n [pairs]” refers to the number of paired comparisons. If multiple comparisons were present in a study, n [pairs] = (n [total] / # of comparisons).

**Supplementary Figure 2.** Summary of evidence search and selection process for the effect of oat β-glucan on postprandial glucose and insulin responses.


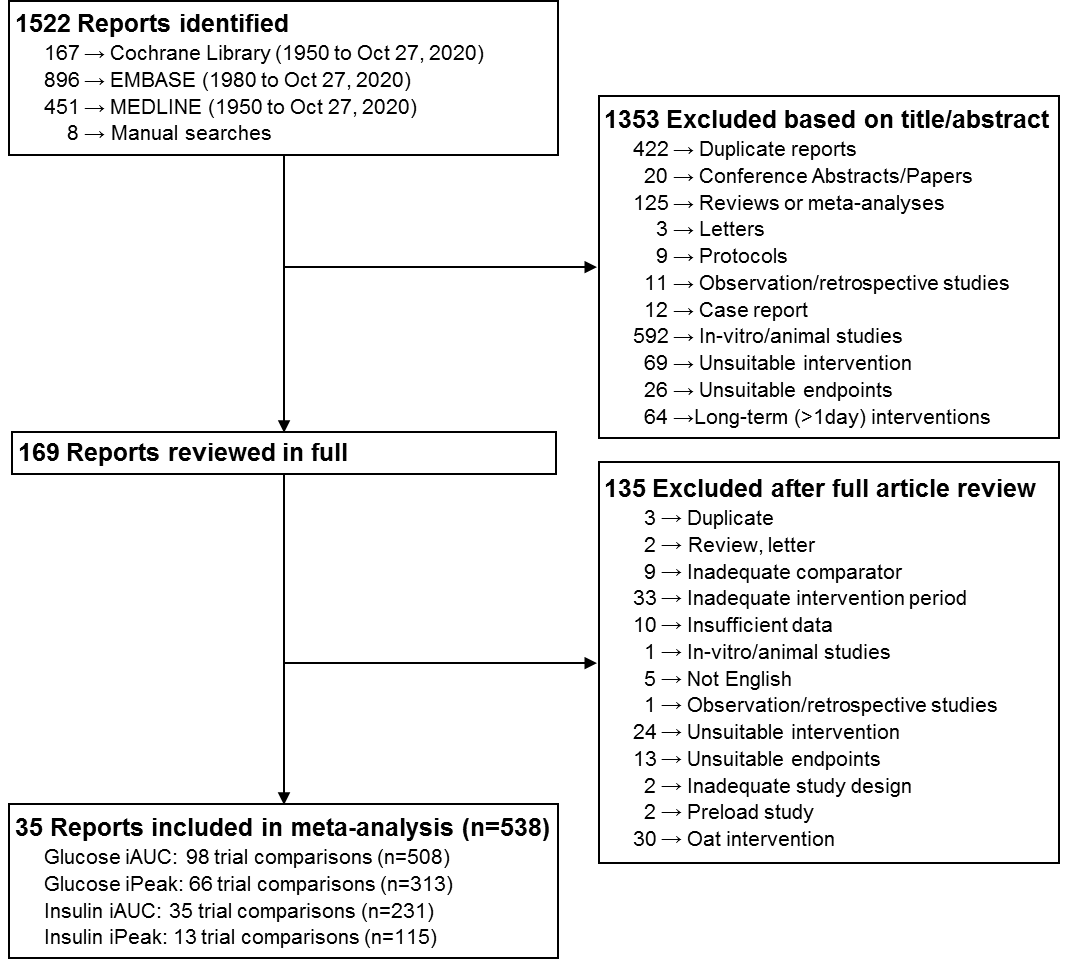


iAUC = incremental area under the curve; iPeak = incremental peak rise.

# **Supplementary Table 3.** Study Characteristics

| **Study, Year**  **[reference]** | **Participants** | **Health Status** | **Age, years** | **BMI, kg/m^2^** | **Intervention** | | | | **Comparator** | | **Duration, mins** | **Setting (OP/IP)** | **Funding** | **Outcome(s)** |
| --- | --- | --- | --- | --- | --- | --- | --- | --- | --- | --- | --- | --- | --- | --- |
|  |  |  |  |  | **Dose, g*** | **avCHO,**  **g†** | **MW,**  **g/mol**‡ | **Description** | **avCHO,**  **g†** | **Description** |  |  |  |  |
| Amiruddin et al. 2019 [73] | 10 (0M, 10F) | Healthy | 22.2+2.0 | 20.6+0.8 | 4 | 50 | - | Chocolate-flavoured drink + OBG | 50 | Control drink | 120 | Malaysia  (OP) | Agency | Glucose iAUC |
|  |  |  |  |  | 4 | 50 | - | Chocolate-flavoured drink + whey protein (5g) + OBG | 50 | Chocolate-flavoured drink + whey protein (5g) |  |  |  |  |
| Beck et al. 2009 [44] | 14  (7M, 7F) | OW | 38.7 | 29.6 | 2.16 | 43.2 | 1,681,000 | Cereal with low-dose OBG sourced from oat bran (Oatwell LBG) | 43.6 | Corn-based control cereal | 240 | Australia (OP) | Industry | Insulin iAUC§ |
|  |  |  |  |  | 3.82 | 42.9 | 1,378,000 | Cereal with mid-dose OBG sourced from oat bran (Oatwell MBG) |  |  |  |  |  |  |
|  |  |  |  |  | 5.45 | 42.6 | 1,213,000 | Cereal with high-dose OBG sourced from oat bran (Oatwell HBG)) |  |  |  |  |  |  |
|  |  |  |  |  | 5.65 | 43.3 | 1,222,000 | Cereal with high-dose OBG source from OBG concentrate (HBGX) |  |  |  |  |  |  |
| Behall et al. 2006 [45] | 20  (0M, 20F) | Mixed  (Healthy  & OW) | 43.4 | 26.2 | 0.3 | 72 | - | Muffin (Low-dose OBG, low-dose RS) | 72 | Glucose solution | 240 | USA  (OP) | - | Glucose iAUC,  Insulin iAUC |
|  |  |  |  |  | 0.3 | 72 | - | Muffin (Low-dose OBG, med-dose RS) |  |  |  |  |  |  |
|  |  |  |  |  | 0.3 | 72 | - | Muffin (Low-dose OBG, high-dose RS) |  |  |  |  |  |  |
|  |  |  |  |  | 0.9 | 72 | - | Muffin (Med-dose OBG, low-dose RS) |  |  |  |  |  |  |
|  |  |  |  |  | 0.9 | 72 | - | Muffin (Med-dose OBG, med-dose RS) |  |  |  |  |  |  |
|  |  |  |  |  | 0.9 | 72 | - | Muffin (Med-dose OBG, high-dose RS) |  |  |  |  |  |  |
|  |  |  |  |  | 3.7 | 72 | - | Muffin (High-dose OBG, low-dose RS) |  |  |  |  |  |  |
|  |  |  |  |  | 3.7 | 72 | - | Muffin (High-dose OBG, med-dose RS) |  |  |  |  |  |  |
|  |  |  |  |  | 3.7 | 72 | - | Muffin (High-dose OBG, high-dose RS) |  |  |  |  |  |  |
| Binou et al. 2020 [74] | 10 (5M, 5F) | Healthy | 27.0+3.9 | 24.5+2.8 | 6 | 50 | - | Wheat bread enriched with OBG | 50 | 50g glucose in 250mL water | 180 | Greece  (OP) | Industry | Glucose iAUC |
| Braaten et al. 1991 [46] | 10  (4M, 6F) | Healthy | 25.0+5.8 | 24.9+4.3 | 11.3 | 51.1 | 804,950ƒ | Gel-like pudding containing 50g glucose + 14.5g oat gum | 50 | 50g glucose in 500mL water | 180 | Canada  (OP) | Industry | Glucose iAUC, Glucose iPeak |
| Braaten et al. 1994 [47] | 11  (7M, 4F) | Healthy | 51.9+6.2 | 27.3+2.0 | 8.8 | 60.7 | 900,000¶ | Oat gum + wheat farina | 62.2 | Wheat farina | 180 | Canada  (OP) | Agency & Industry | Glucose iAUC, Insulin iAUC, Glucose iPeak |
|  |  |  |  |  | 8.8 | 56.6 | - | Oat Bran |  |  |  |  |  |  |
|  | 10  (7M, 3F) | T2D | 58.4+5.5 | 27.7+3.0 | 8.8 | 60.7 | 900,000¶ | Oat gum + wheat farina | 62.2 | Wheat farina | 180 |  |  |  |
|  |  |  |  |  | 8.8 | 56.6 | - | Oat Bran |  |  |  |  |  |  |
| Brennan et al. 2012 [48] | 12  (4M, 8F) | Healthy | Range: 18-40 | Range: 22.5-28 | 0.21 | 25 | - | 15% oat bran substitution with wheat flour in ready-to-eat snack product | 25 | Ready-to-eat snack product without oat bran | 120 | UK  (OP) | - | Glucose iAUC |
| Brummer et al. 2012 [49] | 12  (3M, 9F) | Healthy | 48.5+1.9 | 28+2.0 | 8.6 | 31 | 2,180,000 | Oat bran cereal (3H) | 31 | White bread | 120 | Canada  (OP) | Agency | Glucose iAUC, Glucose iPeak |
|  |  |  |  |  | 8.3 | 31 | 921,000 | Oat bran cereal (4M) |  |  |  |  |  |  |
|  |  |  |  |  | 8.7 | 31 | 627,000 | Oat bran cereal (3M) |  |  |  |  |  |  |
|  |  |  |  |  | 8.4 | 31 | 326,000 | Oat bran cereal (4L) |  |  |  |  |  |  |
| Galvao Candino et al. 2015 [50] | 10  (5M, 5F) | Healthy | 22.9+1.7 | 22.1+2.0 | 2.75 | 75 | - | Oat Bran Shake (50g oat bran, 3.71g powdered skim milk, 15g cocoa powder, 41.8g maltodextrin, 12.4g soybean oil, 20 drops sweetener) | 75 | Control Shake (23.7g powdered skim milk, 15g cocoa powder, 58.5g maltodextrin, 15.4g soybean oil, 20 drops sweetener) | 120 | Brazil  (OP) | - | Glucose iAUC |
| Grandfeldt et al. 2008 [51] – series 1 | 19  (6M, 13F) | Healthy | 37.5+15.2 | 22.4+0.6 | 3.0 | 50 | - | Test meal (muesli with flakes made from oat bran (OatWell) + yogurt + sandwich) | 48.5 | Reference meal (yogurt + sandwich) | 120 | Sweden  (OP) | Industry | Glucose iAUC,  Insulin iAUC |
| Grandfeldt et al. 2008 [51] – series 2 | 13  (5F, 8M) | Healthy | 37.5+3.6 | 22.4+0.6 | 4.0 | 50 | - | Test meal (muesli with flakes made from oat bran (OatWell) + yogurt + sandwich) | 50 | Reference meal (yogurt + sandwich) |  |  |  |  |
| Hartvigsen et al. 2014 [52] | 15  (7M, 8F) | MetS | 62.8+4.2 | 31.1+3.2 | 4.2 | 50 | 1,978,000 | Wheat bread + concentrated OBG (PromOat) | 50 | Wheat Bread | 120 | Denmark (OP) | Agency | Glucose iAUC,  Insulin iAUC |
| Hlebowicz et al. 2008 [53] | 12  (8M, 4F) | Healthy | 27+5 | 22+3 | 4.0 | 32.7 | - | Muesli with flakes made from oat bran (OatWell) | 38.7 | Muesli containing cornflakes | 60 | Sweden (OP) | Industry | Glucose iAUC |
| Holm et al. 1992 -AJCN [54] | 10  (3M, 7F) | Healthy | Range:  M, 34-44; F, 26-49 | Range:  18.5-23.5;  17.5-23.0 | 8.6 | 50 | - | Oat bran bread  (HSFB-ob) | 50 | White wheat bread  (WWB-tl) | 180 | Sweden  (OP) | Industry | Glucose iAUC,  Insulin iAUC |
| Holm et al. 1992 -EJCN [55] | 10  (9M, 1F) | Healthy | Range: 23-45 | Range: 18-23.1 | 5.2 | 54.2 | - | Oat bran pasta | 54.2 | Durum pasta | 180 | Switzerland  (OP) | Agency | Glucose iAUC,  Insulin iAUC,  Glucose iPeak,  Insulin iPeak |
| Jenkins et al. 2002 [56] | 16  (10M, 6F) | T2D | 61+7.2 | 29+6.4 | 7.3 | 50 | - | OBG-enriched breakfast cereal | 50 | White bread | 180 | Canada  (OP) | Industry | Glucose iAUC |
|  |  |  |  |  | 6.2 | 50 | - | OBG-enriched snack bar |  |  |  |  |  |  |
|  |  |  |  |  | 3.7 | 50 | - | Oat bran breakfast cereal |  |  |  |  |  |  |
| Juntunen et al. 2002 [57] | 20 (10M,10F) | Healthy | 28.5+5.6 | 22.9+2.9 | 5.4 | 50 | 250,000 | OBG-enriched rye bread | 50 | White wheat bread | 180 | Finland  (OP) | Agency & Industry | Glucose iPeak, Insulin iPeak |
| Kwong et al. 2013 – Food & Function [58] | 15  (7M, 8F) | Healthy | 37.2+11.2 | 26.6+4.3 | 4.0 | 50 | 145,000 | 4g Low MW OBG + 50g dextrose Drink (LD) | 50 | 50g dextrose drink without OBG (ND) | 120 | Canada  (OP) | Agency | Glucose iAUC, Glucose iPeak |
|  |  |  |  |  | 4.0 | 50 | 580,000 | 4g High MW OBG + 50g dextrose Drink (HD) |  |  |  |  |  |  |
|  |  |  |  |  | 4.0 | 50 | 145,000 | 4g Low MW OBG + 50g dextrose Gel (4LG) |  |  |  |  |  |  |
|  |  |  |  |  | 4.0 | 50 | 362,500 | 2g High MW OBG + 2g Low MW OBG + 50g dextrose Gel (2H2LG) |  |  |  |  |  |  |
|  |  |  |  |  | 4.0 | 50 | 471,250 | 3g High MW OBG + 1g Low MW OBG + 50g dextrose Gel (3H1LG) |  |  |  |  |  |  |
| Kwong et al. 2013 - BJN [59] | 15  (7M, 8F) | Healthy | 37.2+11.2 | 26.6+4.3 | 4.0 | 50 | 145,000 | 250mL 50g glucose solution with 4g Low MW OBG (250L) | 50 | 250mL 50g glucose solution (250N) | 120 | Canada  (OP) | Agency | Glucose iAUC, Glucose iPeak |
|  |  |  |  |  | 4.0 | 50 | 580,000 | 250mL 50g glucose solution with 4g High MW OBG (250H) |  |  |  |  |  |  |
|  |  |  |  |  | 4.0 | 50 | 145,000 | 600mL 50g glucose solution with 4g Low MW OBG (600L) | 50 | 600mL 50g glucose solution (600N) |  |  |  |  |
|  |  |  |  |  | 4.0 | 50 | 580,000 | 600mL 50g glucose solution with 4g High MW OBG (600H) |  |  |  |  |  |  |
| Lad-Pidhainy et al. 2007 [60] | 11  (4M, 7F) | Healthy | 34.6+10.4 | 24.7+4.8 | 7.6 | 50 | 2,800,000 | Oat bran muffin with 8g OBG  (8-g Fresh) | 50 | Whole Wheat Muffin (Control) | 120 | Canada  (OP) | - | Glucose iAUC, Glucose iPeak |
|  |  |  |  |  | 7.6 | 50 | 2,000,000 | Oat bran muffin with 8g OBG + 2 freeze thaw temperature cycling (8-g 2FT) |  |  |  |  |  |  |
|  |  |  |  |  | 7.6 | 50 | 1,800,000 | Oat bran muffin with 8g OBG + 4 freeze thaw temperature cycling (8-g 4FT) |  |  |  |  |  |  |
|  |  |  |  |  | 10.8 | 50 | 2,700,000 | Oat bran muffin with 12g OBG  (12-g Fresh) |  |  |  |  |  |  |
|  |  |  |  |  | 10.8 | 50 | 2,400,000 | Oat bran muffin with 12g OBG + 2 freeze thaw temperature cycling (12-g 2FT) |  |  |  |  |  |  |
|  |  |  |  |  | 10.8 | 50 | 2,000,000 | Oat bran muffin with 12g OBG + 4 freeze thaw temperature cycling (12-g 4FT) |  |  |  |  |  |  |
| Lindstrom et al. 2015 [61] | 18  (8M, 10F) | OW | 48.1±15.6 | 27.9±2.4 | 3.0 | 50 | - | Liquid oat bran mixed with cow’s milk | 50 | White wheat bread | 180 | Sweden  (OP) | Agency | Glucose iAUC, Insulin iAUC,  Glucose iPeak |
| Panahi et al. 2007 [62] | 11  (5M, 6F) | Healthy | 34+5 | 23.0+0.8 | 6 | 75 | - | 75g-OGTT + 11.05g Oat-A (concentrate obtained by an alcohol-based enzymatic process) | 75 | 75g-OGTT + 8g fructose oligosaccharide + 3g wheat bran (control) | 120 | Canada  (OP) | Agency | Glucose iAUC |
|  |  |  |  |  | 6 | 75 | - | 75g-OGTT + 10.17g Oat-B (concentrate obtained by an aqueous extraction process) | 75 |  |  |  |  |  |
| Panahi et al. 2014 [63] | 12  (7M, 5F) | Healthy | 26.1±6.9 | 24.8±3.1 | 1.5 | 50 | - | OBG-enriched snack bar from oat concentrate | 50 | Snack bar without OBG | 120 | Canada  (OP) | Agency & Industry | Glucose iAUC |
|  |  |  |  |  | 3.0 | 50 | - | OBG-enriched snack bar from oat concentrate |  |  |  |  |  |  |
|  |  |  |  |  | 6.0 | 50 | - | OBG-enriched snack bar from oat concentrate |  |  |  |  |  |  |
| Paquin et al. 2013 [64] | 14  (14M, 0F) | Healthy | 32±9 | 23.9±4 | 1.1 | 35 | - | Fruit juice enriched with OBG | 35 | Fruit Juice without OBG | 120 | Canada  (OP) | Agency & Industry | Glucose iAUC, Insulin iAUC,  Glucose iPeak,  Insulin iPeak |
| Regand et al. 2009 [65] | 12  (6M, 6F) | Healthy | 42.3±14.6 | 28.8±5.5 | 4.0 | 64 | 197,000 | Oat crisp bread | 65 | Wheat crisp bread | 120 | Canada  (OP) | Agency | Glucose iAUC, Glucose iPeak |
|  |  |  |  |  | 4.0 | 43 | 1,942,000 | Oat porridge | 46 | Whole wheat muffin |  |  |  |  |
|  |  |  |  |  | 4.0 | 44 | 1,911,000 | Oat granola |  |  |  |  |  |  |
|  |  |  |  |  | 4.0 | 42 | 465,000 | Oat pasta | 44 | Whole wheat pasta |  |  |  |  |
| Regand et al. 2011 [66] | 12  (6M, 6F) | Healthy | 27.3±5.5 | 25.6±5.0 | 6.2 | 38 | 57,000 | Oat Granola  (Low MW OBG, 40g avCHO) | 40 | Wheat granola  (40g avCHO) | 120 | Canada  (OP) | Agency | Glucose iAUC, Glucose iPeak |
|  |  |  |  |  | 6.2 | 38 | 435,000 | Oat Granola  (Med MW OBG, 40g avCHO) |  |  |  |  |  |  |
|  |  |  |  |  | 6.2 | 38 | 2,133,000 | Oat Granola  (High MW OBG, 40g avCHO) |  |  |  |  |  |  |
|  |  |  |  |  | 6.3 | 60 | 82,000 | Oat Granola  (Low MW OBG, 60g avCHO) | 58 | Wheat granola  (60g avCHO) |  |  |  |  |
|  |  |  |  |  | 6.3 | 60 | 325,000 | Oat Granola  (Med MW OBG, 60g avCHO) |  |  |  |  |  |  |
|  |  |  |  |  | 6.3 | 60 | 1,996,000 | Oat Granola  (High MW OBG, 60g avCHO) |  |  |  |  |  |  |
| Rieder et al. 2019 [67] | 14 | Healthy | 44.8±13.8 | 24.3±1.7 | 3.8 | 26.2 | 282,000 | Degraded oat bran concentrate bread (Degraded OBCB) | 26 | White wheat bread | 120 | UK  (OP) | Agency | Glucose iAUC, Glucose iPeak |
|  |  |  |  |  | 3.8 | 26.3 | 592,000 | Optimal oat bran concentrate bread (Optimal OBCB) |  |  |  |  |  |  |
|  |  |  |  |  | 1.7 | 26.4 | 421,000 | Low oat bran concentrate bread (Low OBCB) |  |  |  |  |  |  |
| Tapola et al. 2005 [68] | 12  (7M, 5F) | T2D | 66±7 | 28.9±3.5 | 9.4 | 12.5 | - | Oat bran flour (61.6g) mixed with cold water | 12.5 | 12.5g glucose drink | 120 | Finland  (OP) | - | Glucose iAUC |
|  |  |  |  |  | 3.0 | 12.5 | - | Cold water poured onto oat bran crisp (29.1g) |  |  |  |  |  |  |
|  |  |  |  |  | 4.6 | 31.1 | - | 25g glucose drink with oat bran flour (30g) | 25 | 25g glucose drink |  |  |  |  |
| Tappy et al. 1996 [69] | 8  (7M, 1F) | T2D | 56.1±10.4 | 29.6±5.3 | 4.0 | 35 | - | OBG-enriched breakfast cereal | 35 | Standard continental breakfast | 240 | Switzerland  (OP) | - | Glucose iAUC, Glucose iPeak |
|  |  |  |  |  | 6.0 | 35 | - | OBG-enriched breakfast cereal |  |  |  |  |  |  |
|  |  |  |  |  | 8.4 | 35 | - | OBG-enriched breakfast cereal |  |  |  |  |  |  |
| Tosh et al. 2008 [7] | 10  (6M, 4F) | Healthy | 37.6+18.0 | 23.8+4.4 | 4.4 | 47.9 | 130,000 | Oat bran muffins prepared with enzyme to get low MW OBG (4g L) | 51.4 | Whole wheat muffin | 120 | Canada  (OP) | Agency | Glucose iAUC, Glucose iPeak |
|  |  |  |  |  | 4.4 | 47.9 | 380,000 | Oat bran muffins prepared with enzyme to get medium MW OBG (4g M) |  |  |  |  |  |  |
|  |  |  |  |  | 4.4 | 47.9 | 590,000 | Oat bran muffins prepared with enzyme to get high MW OBG (4g H) |  |  |  |  |  |  |
|  |  |  |  |  | 4.4 | 47.9 | 2,190,000 | Oat bran muffins prepared without enzyme (4g N) |  |  |  |  |  |  |
|  |  |  |  |  | 8.2 | 45 | 220,000 | Oat bran muffins prepared with enzyme to get low MW OBG (8g L) |  |  |  |  |  |  |
|  |  |  |  |  | 8.2 | 45 | 410,000 | Oat bran muffins prepared with enzyme to get medium MW OBG (8g M) |  |  |  |  |  |  |
|  |  |  |  |  | 8.2 | 45 | 760,000 | Oat bran muffins prepared with enzyme to get high MW OBG (8g H) |  |  |  |  |  |  |
|  |  |  |  |  | 8.2 | 45 | 2,230,000 | Oat bran muffins prepared without enzyme (8g N) |  |  |  |  |  |  |
| Ulmius et al. 2011 [70] | 15  (8M, 7F) | Healthy | Range:  20-28 | 22.8±2.1 | 4.5 | 75 | - | Oat bran (82g), black currant beverage with pulp (250g), white bread (15.5g) | 75 | Black currant beverage with pulp (250g), white bread (67.7g), dextrose (10g), rapeseed oil (5.9g) | 180 | Sweden  (OP) | Agency | Glucose iAUC, Insulin iAUC, Glucose iPeak, Insulin iPeak |
| Wang et al. 2017 [75] | 10 (5M, 5F) | Healthy | 25.2+2.0 | 22.4+1.2 | 1.25 | 25 | - | OBG-enriched steamed bread | 25 | Steamed bread without OBG (Control) | 120 | China  (OP) | Agency | Glucose iAUC |
| Wolever et al. 2018 [6] | 40  (22M, 18F) | Healthy | 31.5±11.0 | 25.1±3.0 | 1.4 | 27.7 | - | Instant oatmeal + 0.72g oat bran | 27.6 | Instant oatmeal without oat bran | 120 | Canada  (OP) | Industry | Glucose iAUC, Glucose iPeak |
|  |  |  |  |  | 1.6 | 27.7 | - | Instant oatmeal + 1.43g oat bran |  |  |  |  |  |  |
|  |  |  |  |  | 2.0 | 27.9 | - | Instant oatmeal + 2.86g oat bran |  |  |  |  |  |  |
|  |  |  |  |  | 2.8 | 28.1 | - | Instant oatmeal + 5.72g oat bran |  |  |  |  |  |  |
| Wolever et al. 2020 [76] | 28  (16M, 12F) | Healthy | 33.1+10.6 | 24.8+2.3 | 2 | 51.2 | 1,999,000 | Instant oatmeal (27g) + oat bran (3g) + standard breakfast (25-27g white bread, 5-7g butter, 11-21g jam, 240mL 2% milk) | 51.3 | Cream of Rice | 180 | Canada (OP) | Industry | Glucose iAUC, Insulin iAUC, Glucose iPeak, Insulin iPeak |
|  |  |  |  |  | 4 | 51.2 | 2,060,000 | Instant oatmeal (27g) + oat bran (10.1g) + standard breakfast (25-27g white bread, 5-7g butter, 11-21g jam, 240mL 2% milk) |  |  |  |  |  |  |
|  |  |  |  |  | 4 | 51.2 | <10,000 | Instant oatmeal (27g) + oat bran (10.1g) treated with β-glucanase + standard breakfast (25-27g white bread, 5-7g butter, 11-21g jam, 240mL 2% milk) |  |  |  |  |  |  |
| Wood et al. 1990 [71] | 9  (4M, 5F) | Healthy | 23.6+3.9 | 24.5+4.2 | 11.7 | 50 | 900,000¶ | Glucose drink (50g) + 14.5g oat gum | 50 | Glucose drink (50g) without oat gum | 180 | Canada  (OP) | Industry | Glucose iAUC |
|  | 10  (7M, 3F) | Healthy | 52+7.3 | 27.3+11.7 | 8.9 | 60 | 900,000¶ | Cream of wheat (48g) + 11g oat gum | 60 | Cream of wheat control (68g) without oat gum |  |  |  |  |
| Wood et al. 1994 [72] | 9  (4M, 5F) | Healthy | 31.4+4.9 | 24.8+6.2 | 1.5 | 50 | 804, 950ƒ | 50g glucose drink + 1.8g oat gum | 50 | 50g glucose drink without oat gum | 180 | Canada  (OP) | Industry | Glucose iAUC, Insulin iAUC, Glucose iPeak, Insulin iPeak |
|  |  |  |  |  | 2.9 | 50 | 804, 950ƒ | 50g glucose drink + 3.6g oat gum |  |  |  |  |  |  |
|  |  |  |  |  | 5.8 | 50 | 804, 950ƒ | 50g glucose drink + 7.2g oat gum |  |  |  |  |  |  |
|  | 11  (6M, 5F) | Healthy | 34.3+10.4 | 24.3+2.5 | 6.4 | 50 | 250,800ƒ | 50g glucose drink + 7.2g oat gum acid-hydrolyzed for 15mins (OG15) | 50 | 50g glucose drink without oat gum |  |  |  |  |
|  |  |  |  |  | 6.4 | 50 | 101,850ƒ | 50g glucose drink + 7.2g oat gum acid-hydrolyzed for 60mins (OG60) |  |  |  |  |  |  |
|  | 8  (6M, 2F) | Healthy | 39.8+14.3 | 26.4+1.9 | 5.8 | 50 | - | 36.2g Instagum (containing 7.2g oat gum and 29g DE 10 maltodextrin), 250ml degassed diet soft drink (7-Up), 250ml degassed soda water containing 21g glucose | 53 | Control drink (21g glucose + 29g DE 10 maltodextrin) |  |  |  |  |

Footnotes on next page

Data represent mean±SD, unless stated otherwise. “-” represents lack of reporting of data. BMI, body mass index; avCHO, available carbohydrate; MW, molecular weight; OP, outpatient; M, male; F, female; OW, overweight; OBG, oat beta-glucan; netAUC, net area under the curve; iAUC, incremental area under the curve; RS, resistant starch; iPeak, incremental peak; T2D, type 2 diabetes.

* For studies where oat bran was provided and dose was specified (Galvao Candino et al. 2015 [50], Ulmius et al. 2011 [70]), OBG dose was estimated to be 5.5% of the oat bran dose. For Holm et al. 1992 – AJCN [54], as this calculation would yield a larger dose than the soluble dietary fibre reported, OBG dose was calculated by taking the difference of the soluble fibre in OSFB-ob and WWB-mg.

† Behall et al. 2006 [39] – 1g avCHO/kg body weight, average given

‡ Individual study MW categorizations may differ from categories that were used in this systematic review and meta-analysis. In this analysis, low MW OBG were considered to be <300 kg/mol, medium MW were 300 to ≤1,000 kg/mol, and high MW were >1,000 kg/mol.

§ Beck et al. 2009 [38] – netAUC insulin (0-2h) data were extracted and analyzed as iAUC data because insulin levels do not dip below baseline levels at 120mins (Figure 2a).

ƒ Data retrieved from Table 1 (Wood, P., Beer, M., & Butler, G. (2000). Evaluation of role of concentration and molecular weight of oat β-glucan in determining effect of viscosity on plasma glucose and insulin following an oral glucose load. British Journal of Nutrition, 84(1), 19-23. doi:10.1017/S0007114500001185)

¶ Data retrieved from Wood, P.J. (2010), REVIEW: Oat and Rye β‐Glucan: Properties and Function. Cereal Chemistry, 87: 315-330. doi:10.1094/CCHEM-87-4-0315.

# **Supplementary Table 4.** RoB of trial comparisons on the effect of OBG on glucose iAUC. Trial comparisons were assessed as having some concerns in the measurement of the outcome if either the dose of OBG was not specified or the sum of the other study methodology criteria was <7

**Supplementary Table 4** *(continued)*

Green circles, low risk of bias; yellow circles, some concerns; red circles, high risk of bias

# **Supplementary Table 5.** RoB of trial comparisons on the effect of OBG on glucose iPeak. Trial comparisons were assessed as having some concerns in the measurement of the outcome if either the dose of OBG was not specified or the sum of the other study methodology criteria was <6

**Supplementary Table 5** *(continued)*

Green circles, low risk of bias; yellow circles, some concerns; red circles, high risk of bias

# **Supplementary Table 6.** RoB of trial comparisons on the effect of OBG on insulin iAUC. Trial comparisons were assessed as having some concerns in the measurement of the outcome if either the dose of OBG was not specified or the sum of the other study methodology criteria was <7

Green circles, low risk of bias; yellow circles, some concerns; red circles, high risk of bias

# **Supplementary Table 7.** RoB of trial comparisons on the effect of OBG on insulin iPeak. Trial comparisons were assessed as having some concerns in the measurement of the outcome if either the dose of OBG was not specified or the sum of the other study methodology criteria was <6

Green circles, low risk of bias; yellow circles, some concerns; red circles, high risk of bias

# **Supplementary Figure 3.** RoB summary on the effect of OBG on glucose iAUC. .

# **Supplementary Figure 4.** RoB summary on the effect of OBG on glucose iPeak.

# **Supplementary Figure 5**. RoB summary on the effect of OBG on insulin iAUC.

# **Supplementary Figure 6.** RoB summary on the effect of OBG on insulin iPeak.

#
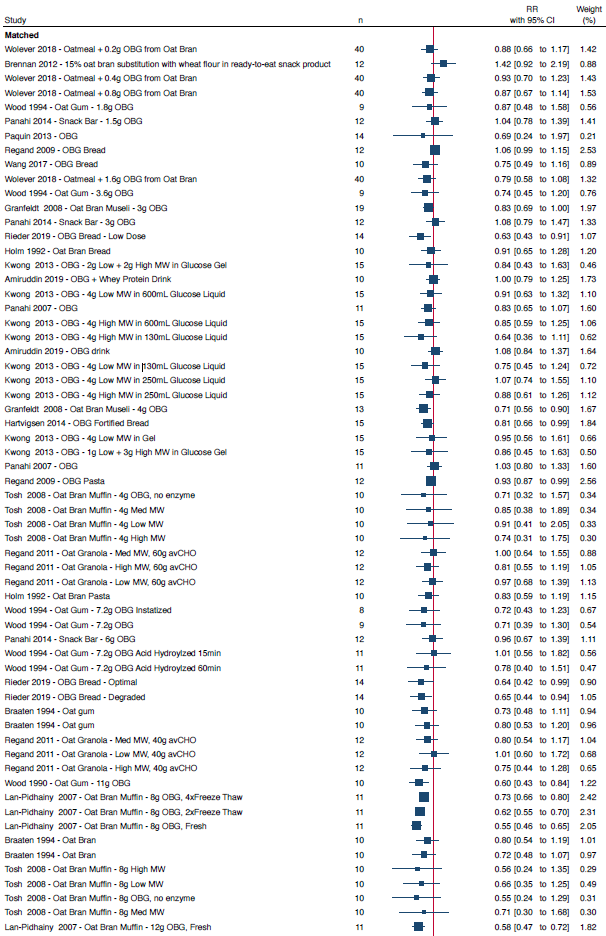
**Supplementary Figure 7**. Pooled effect estimates of OBG and glucose iAUC by type of comparator.


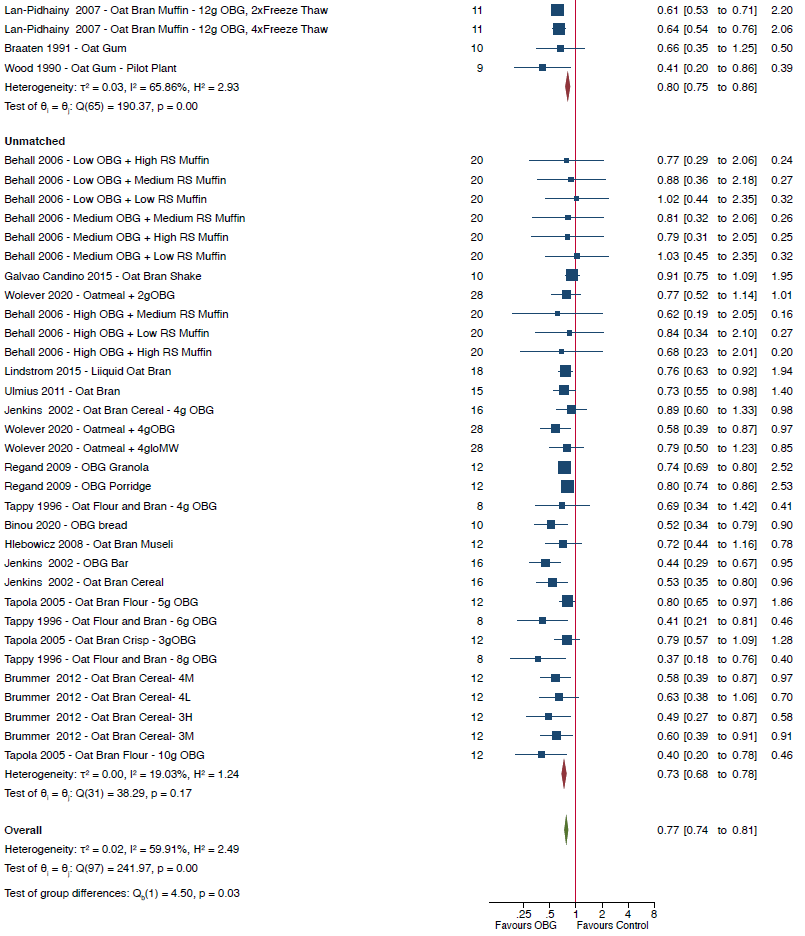
**Supplementary Figure 7** *(continued)*

Data are expressed as ratio of means (RoMs) with 95% CIs using the generic inverse variance method modelled by random effects (DerSimonian-Laird). Trial comparisons within each subgroup are sorted from the lowest to the highest dose of oat β-glucan per 30g available carbohydrate portion The subgroup and total pooled effect estimates are represented by the red and green diamonds, respectively, with the size of the diamond representing the weight of the trial comparison in the overall analysis. Inter-study heterogeneity was assessed using the Cochran Q statistic and quantified using the I^2^ statistic, with P_Q_<0.10 and I^2^>50% considered to be evidence of substantial heterogeneity. Group differences were tested using subgroup meta-analysis where p<0.05 was considered significant.

#
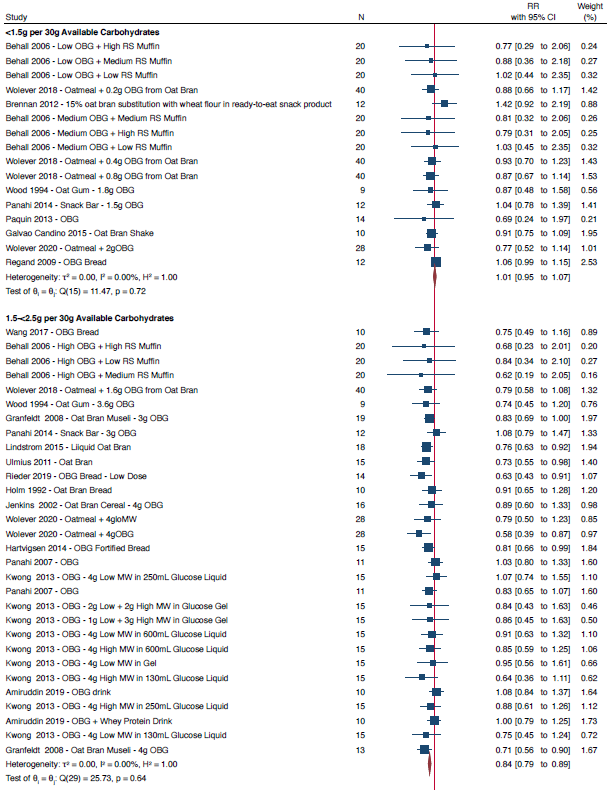
**Supplementary Figure 8.** Pooled effect estimates of OBG dose and glucose iAUC.

**Supplementary Figure 8** *(continued)*


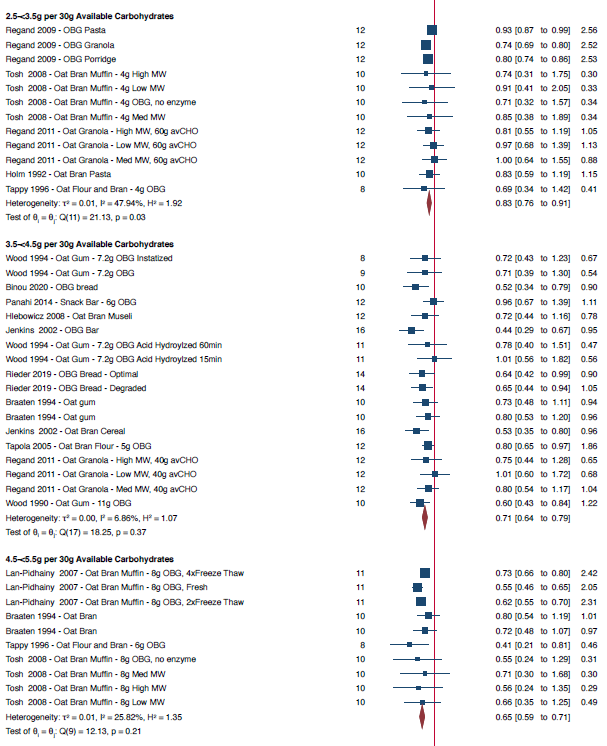


**Supplementary Figure 8** *(continued)*


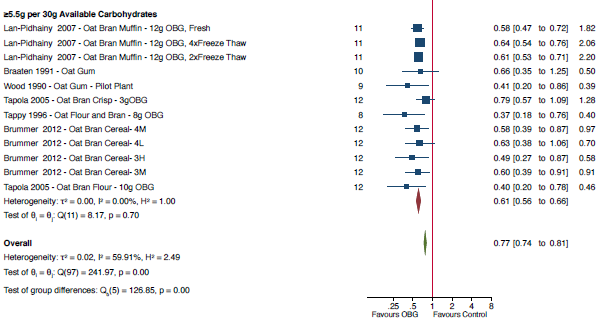


Data are expressed as ratio of means (RoMs) with 95% CIs using the generic inverse variance method modelled by random effects (DerSimonian-Laird). Trial comparisons within each subgroup are sorted from the lowest to the highest dose of oat β-glucan per 30g available carbohydrate portion The subgroup and total pooled effect estimates are represented by the red and green diamonds, respectively, with the size of the diamond representing the weight of the trial comparison in the overall analysis. Inter-study heterogeneity was assessed using the Cochran Q statistic and quantified using the I^2^ statistic, with P_Q_<0.10 and I^2^>50% considered to be evidence of substantial heterogeneity. Group differences were tested using subgroup meta-analysis where p<0.05 was considered significant.

#
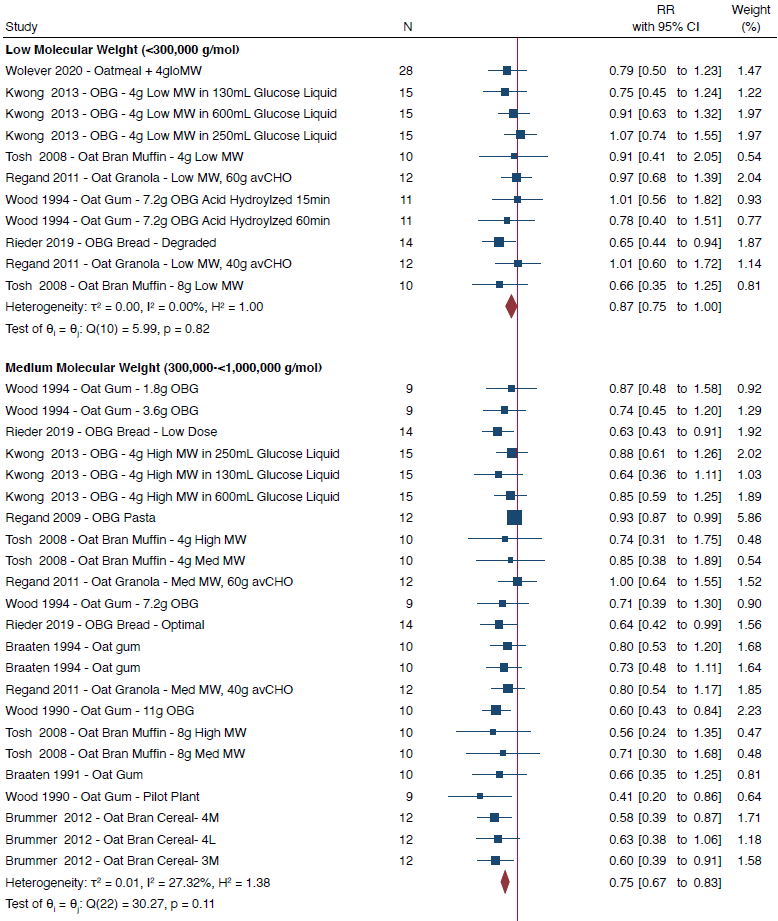
**Supplementary Figure 9.** Pooled effect estimates of OBG molecular weight and glucose iAUC.

**Supplementary Figure 9** *(continued)*


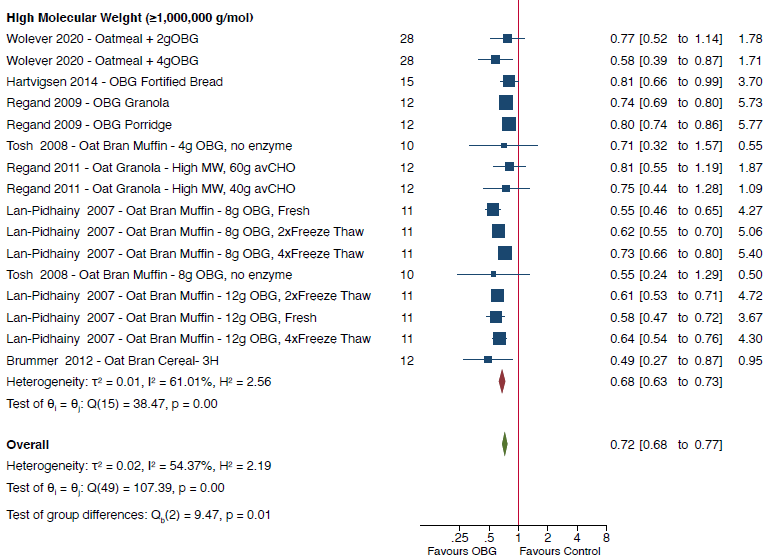


Molecular weight was reported in 47/94 trial comparisons. Data are expressed as ratio of means (RoMs) with 95% CIs using the generic inverse variance method modelled by random effects (DerSimonian-Laird). Trial comparisons within each subgroup are sorted from the lowest to the highest dose of oat β-glucan per 30g available carbohydrate portion The subgroup and total pooled effect estimates are represented by the red and green diamonds, respectively, with the size of the diamond representing the weight of the trial comparison in the overall analysis. Inter-study heterogeneity was assessed using the Cochran Q statistic and quantified using the I^2^ statistic, with P_Q_<0.10 and I^2^>50% considered to be evidence of substantial heterogeneity. Group differences were tested using subgroup meta-analysis where p<0.05 was considered significant.

# **Supplementary Figure 10**. Summary plot of pooled effect estimates of OBG on glucose and insulin iAUC and iPeak by study postprandial duration, intervention food form and study methodology quality.


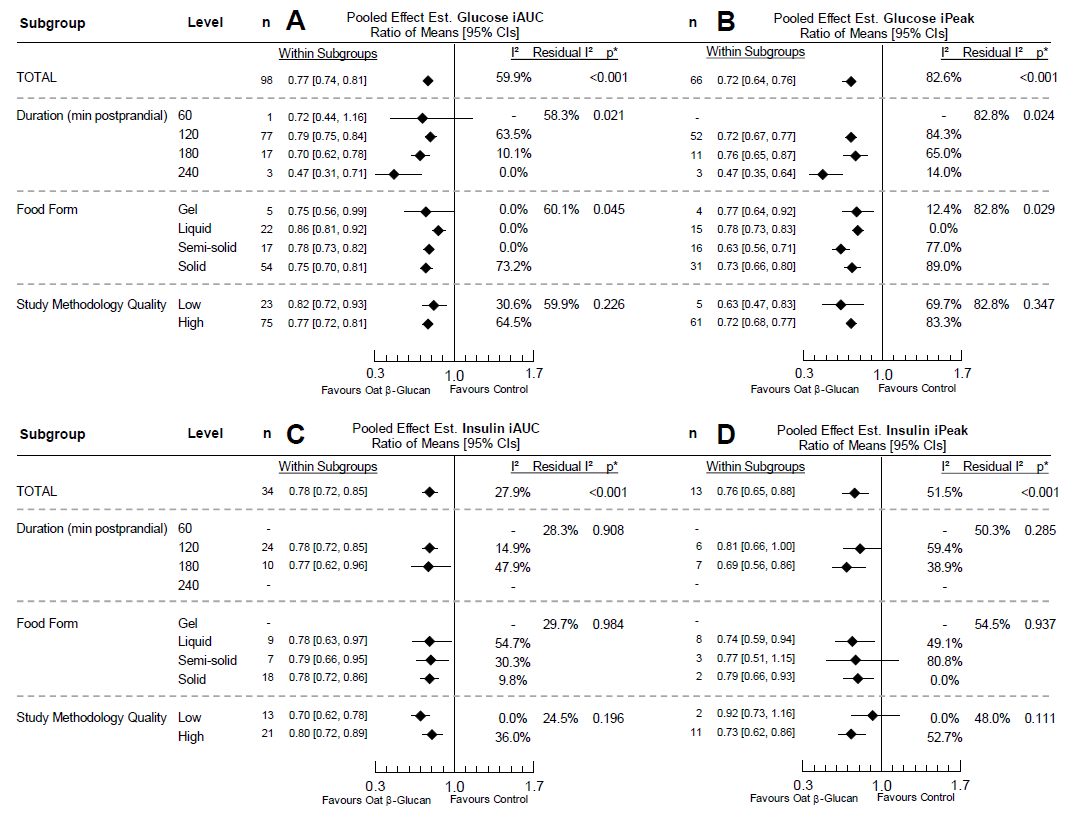


**Panel A**: incremental area under the curve (iAUC) for glucose; **Panel B**, incremental peak rise (iPeak) for glucose; **Panel C**: iAUC for insulin; **Panel D**: iPeak for insulin. Pooled effect estimates are expressed as ratios of means (RoMs, black diamond) with 95% CIs (solid lines). Pooled analyses were conducted using the generic inverse variance method with random effects models. Interstudy heterogeneity was tested by the Cochran Q statistic (χ^2^) at a significance level of P_Q_<0.10 (not shown) and quantified by I^2^. Differences between subgroups were tested using meta-regression and the significance level was reported as a p-value, where p<0.05 was considered significant. The residual I^2^ value represents unexplained heterogeneity for each subgroup. n, number of trial comparisons.

# **Supplementary Figure 11.** Pooled effect estimates of OBG and glucose iAUC by health status.


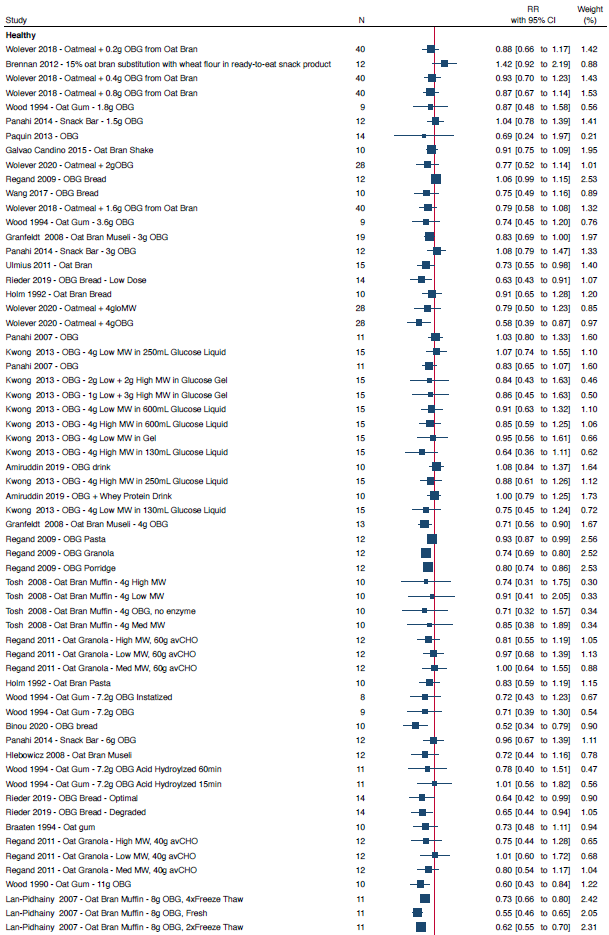


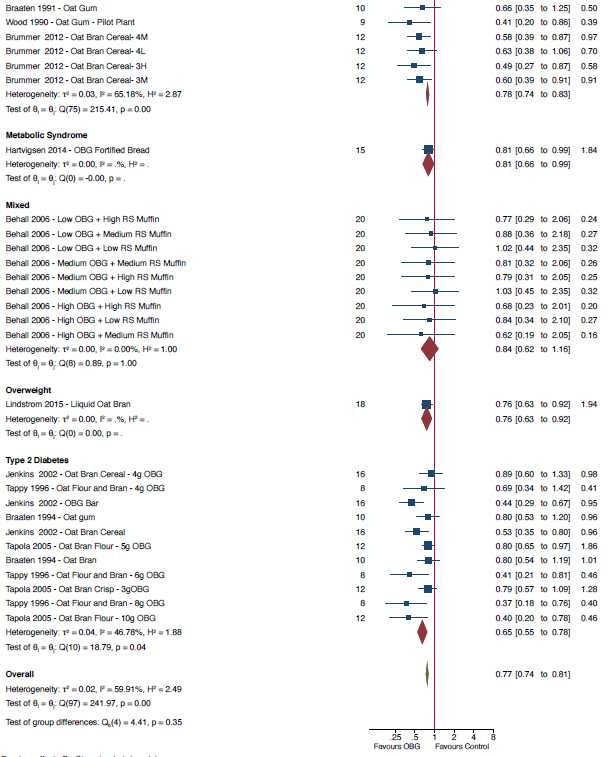
**Supplementary Figure 11** *(continued)*

Data are expressed as ratio of means (RoMs) with 95% CIs using the generic inverse variance method modelled by random effects (DerSimonian-Laird). Trial comparisons within each subgroup are sorted from the lowest to the highest dose of oat β-glucan per 30g available carbohydrate portion The subgroup and total pooled effect estimates are represented by the red and green diamonds, respectively, with the size of the diamond representing the weight of the trial comparison in the overall analysis. Inter-study heterogeneity was assessed using the Cochran Q statistic and quantified using the I^2^ statistic, with P_Q_<0.10 and I^2^>50% considered to be evidence of substantial heterogeneity. Group differences were tested using subgroup meta-analysis where p<0.05 was considered significant.

# **Supplementary Figure 12**. RoB subgroup analysis of OBG on glucose iAUC


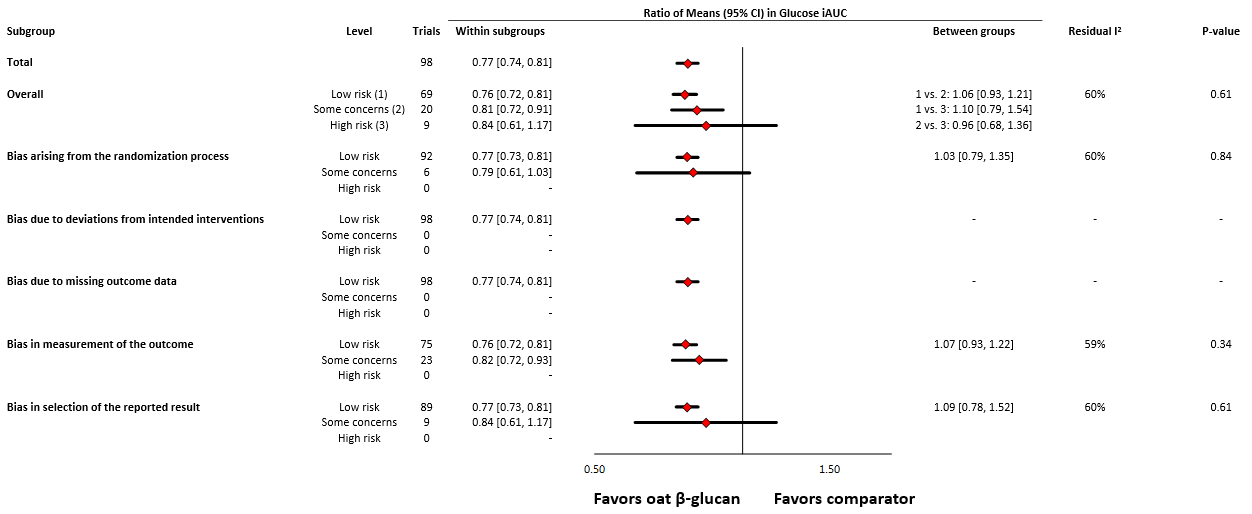


Data are expressed as ratio of means (RoM) with 95% confidence intervals (CIs). Differences between subgroups were tested using meta-regression and the significance level was reported as a p-value, where p<0.05 was considered significant. Pooled effect estimates for each subgroup are represented by the diamonds. The dashed line represents the pooled effect estimate for the overall analysis. The residual I^2^ value represents remaining between-study variation after adjustment for the subgroup.

# **Supplementary Figure 13.** Pooled dose-response relationship (linear and non-linear) between OBG and glucose and insulin iAUC and iPeak.

Individual trial comparisons are represented by the circles, with the weight of the comparison in the overall analysis represented by the size of the circles. The orange solid line represents the linear dose response which was modelled using one-stage random effects with the generic inverse variance and restricted maximum likelihood methods, assuming a linear function. The solid black line and outer black dashed lines represent the non-linear dose response and 95% CIs, respectively, which were modelled with restricted cubic splines with 3 knots (Harrell’s recommended percentiles). Departure from linearity was assessed using the Wald’s test. The following trial comparisons were outliers and not shown in the figure for Glucose iAUC but are included in the pooled dose response: Tapola 2005 (10g OBG intervention. dose: 22.6, RR, 0.40 [95% CI, 0.20 to 0.78]) and Brennan 2012 (dose: 0.25, RR,1.42 [95% CI, 0.92 to 2.19]).

# **Supplementary Figure 14.** Pooled linear dose-response relationship by health status between OBG and glucose iAUC and iPeak.


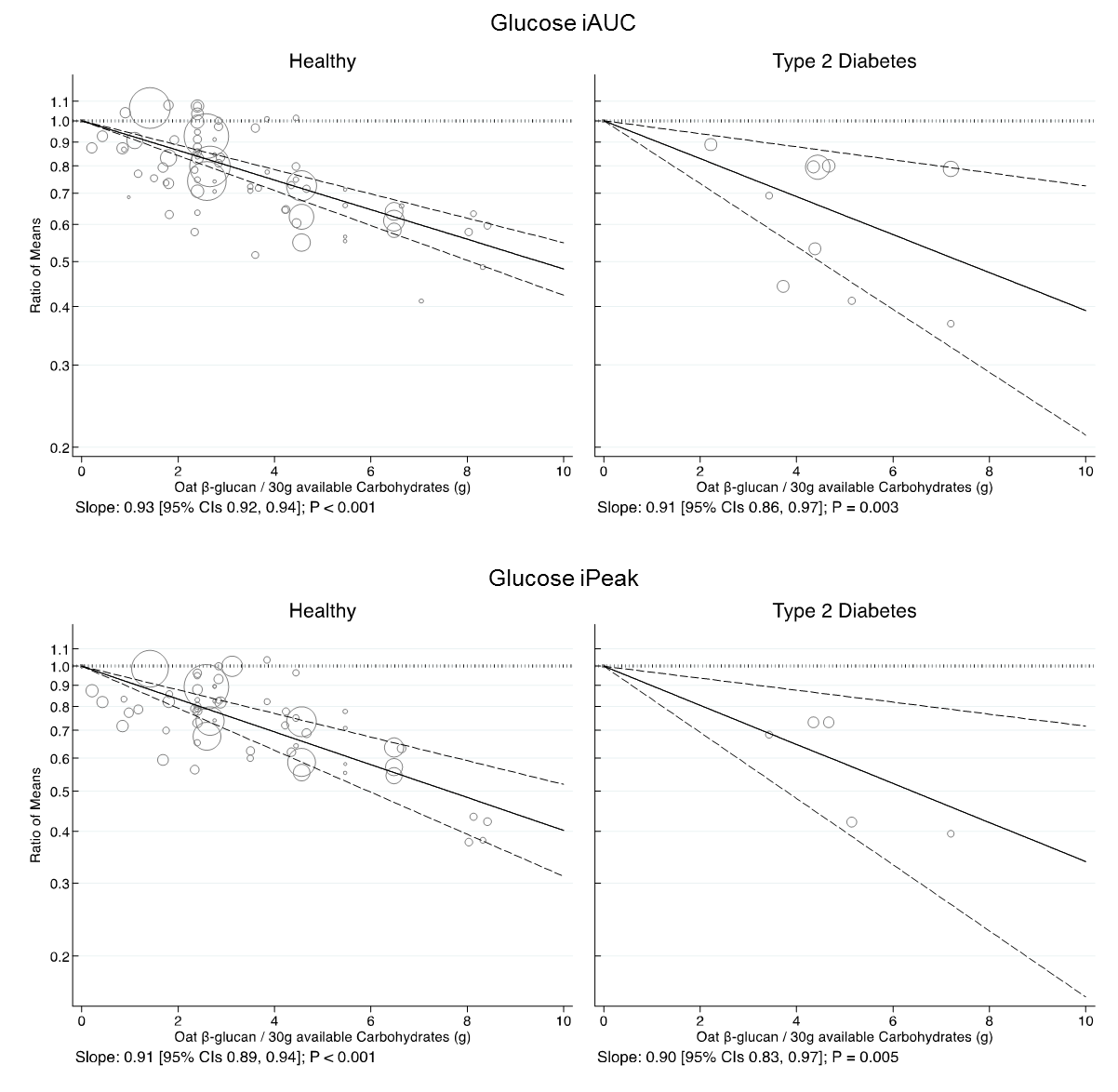


Individual trial comparisons are represented by the circles, with the weight of the comparison in the overall analysis represented by the size of the circles. The fitted dose response estimate is represented by the central solid line with the 95% confidence intervals (CIs) represented by the outer dashed lines, which was modelled using one-stage random effects with the generic inverse variance and restricted maximum likelihood methods, assuming a linear function. The following trial comparisons were outliers and not shown in the figure for Glucose iAUC Healthy and Type 2 Diabetes figures but are included in the pooled dose response: Brennan 2012 (dose: 0.25, RR,1.42 [95% CI, 0.92 to 2.19]) and Tapola 2005 (10g oat β-glucan intervention, dose: 22.6, RR, 0.40 [95% CI, 0.20 to 0.78]), respectively.

# **Supplementary Figure 15.** Pooled linear dose-response relationship by study methodology quality between OBG and glucose and insulin iAUC and iPeak.

Individual trial comparisons are represented by the circles, with the weight of the comparison in the overall analysis represented by the size of the circles. The fitted dose response estimate is represented by the central solid line with the 95% confidence intervals (CIs) represented by the outer dashed lines, which was modelled using one-stage random effects with the generic inverse variance and restricted maximum likelihood methods, assuming a linear function. The following trial comparisons were outliers and not shown in the figure for Glucose iAUC High Quality and Low Quality figures but are included in the pooled dose response: Tapola 2005 (10g oat β-glucan intervention, dose: 22.6, RR, 0.40 [95% CI, 0.20 to 0.78]) and Brennan 2012 (dose: 0.25, RR,1.42 [95% CI, 0.92 to 2.19]), respectively.

# **Supplementary Figure 16.** Pooled linear dose-response relationship by comparator type between OBG and glucose and insulin iAUC and iPeak.

Individual trial comparisons are represented by the circles, with the weight of the comparison in the overall analysis represented by the size of the circles. The fitted dose response estimate is represented by the central solid line with the 95% confidence intervals (CIs) represented by the outer dashed lines, which was modelled using one-stage random effects with the generic inverse variance and restricted maximum likelihood methods, assuming a linear function. The following trial comparisons were outliers and not shown in the figure for Glucose iAUC Matched and Unmatched Comparators figures but are included in the pooled dose response: Brennan 2012 (dose: 0.25, RR,1.42 [95% CI, 0.92 to 2.19]) and Tapola 2005 (10g oat β-glucan intervention, dose: 22.6, RR, 0.40 [95% CI, 0.20 to 0.78]), respectively.

# **Supplementary Figure 17.** Pooled effect estimates of OBG and glucose iPeak by type of comparator.


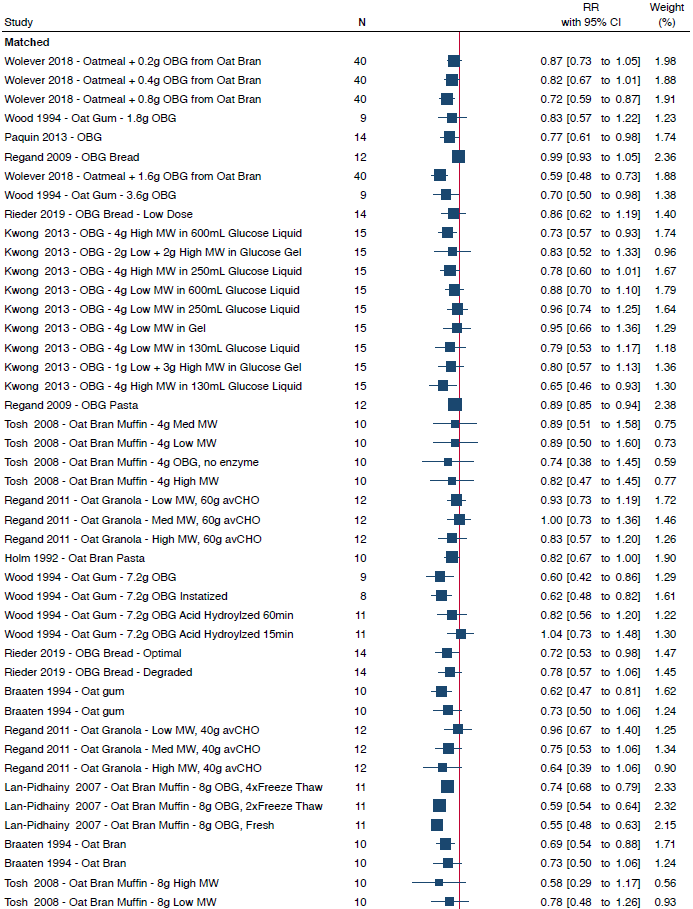


**Supplementary Figure 17** *(continued)*


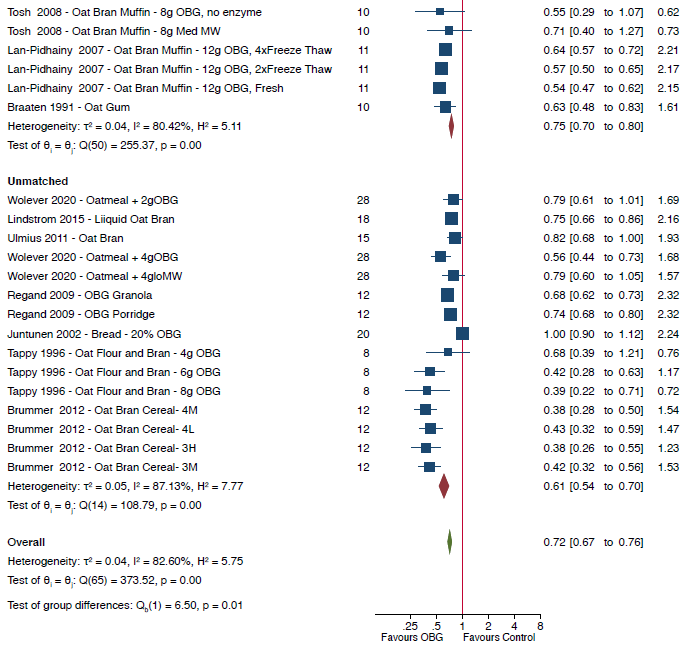


Data are expressed as ratio of means (RoMs) with 95% CIs using the generic inverse variance method modelled by random effects (DerSimonian-Laird). Trial comparisons within each subgroup are sorted from the lowest to the highest dose of oat β-glucan per 30g available carbohydrate portion The subgroup and total pooled effect estimates are represented by the red and green diamonds, respectively, with the size of the diamond representing the weight of the trial comparison in the overall analysis. Inter-study heterogeneity was assessed using the Cochran Q statistic and quantified using the I^2^ statistic, with P_Q_<0.10 and I^2^>50% considered to be evidence of substantial heterogeneity. Group differences were tested using subgroup meta-analysis where p<0.05 was considered significant.

#
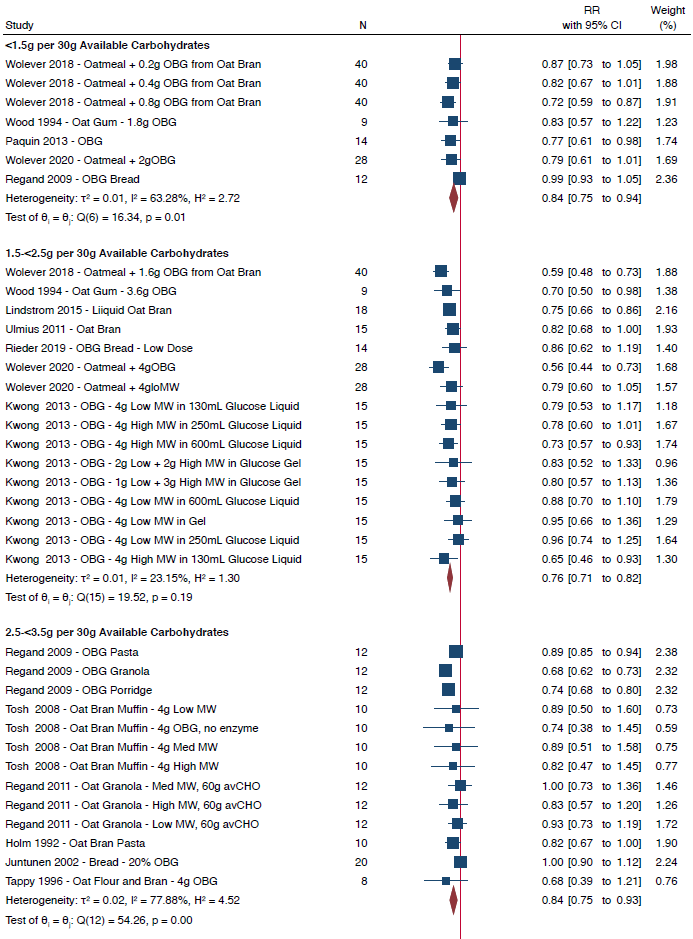
**Supplementary Figure 18.** Pooled effect estimates of OBG dose and glucose iPeak.

**Supplementary Figure 18** *(continued)*


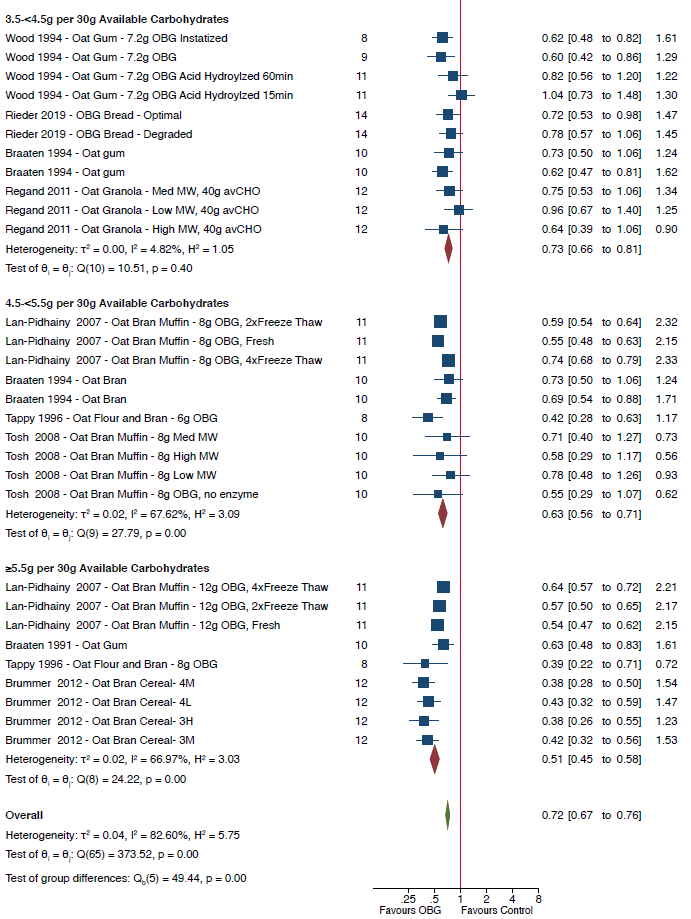


Data are expressed as ratio of means (RoMs) with 95% CIs using the generic inverse variance method modelled by random effects (DerSimonian-Laird). Trial comparisons within each subgroup are sorted from the lowest to the highest dose of oat β-glucan per 30g available carbohydrate portion The subgroup and total pooled effect estimates are represented by the red and green diamonds, respectively, with the size of the diamond representing the weight of the trial comparison in the overall analysis. Inter-study heterogeneity was assessed using the Cochran Q statistic and quantified using the I^2^ statistic, with P_Q_<0.10 and I^2^>50% considered to be evidence of substantial heterogeneity. Group differences were tested using subgroup meta-analysis where p<0.05 was considered significant.

# **Supplementary Figure 19.** Pooled effect estimates of OBG molecular weight and glucose iPeak.


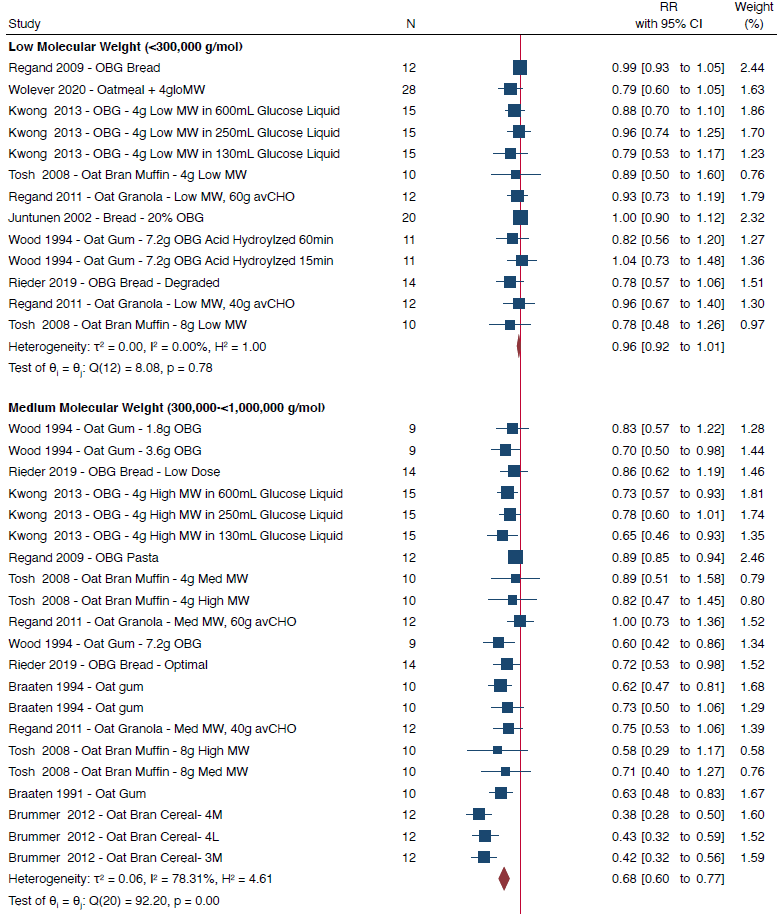


**Supplementary Figure 19** *(continued)*


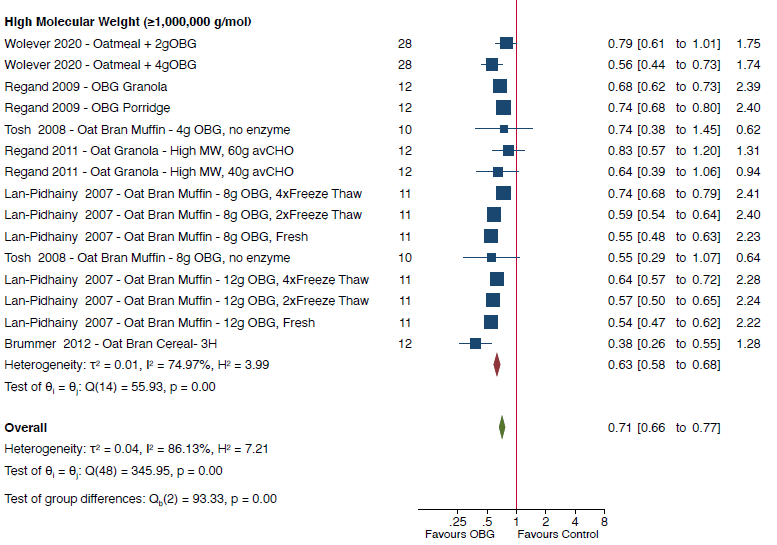


Data are expressed as ratio of means (RoMs) with 95% CIs using the generic inverse variance method modelled by random effects (DerSimonian-Laird). Trial comparisons within each subgroup are sorted from the lowest to the highest dose of oat β-glucan per 30g available carbohydrate portion The subgroup and total pooled effect estimates are represented by the red and green diamonds, respectively, with the size of the diamond representing the weight of the trial comparison in the overall analysis. Inter-study heterogeneity was assessed using the Cochran Q statistic and quantified using the I^2^ statistic, with P_Q_<0.10 and I^2^>50% considered to be evidence of substantial heterogeneity. Group differences were tested using subgroup meta-analysis where p<0.05 was considered significant.

# **Supplementary Figure 20.** Pooled effect estimates of OBG and glucose iPeak by health status.


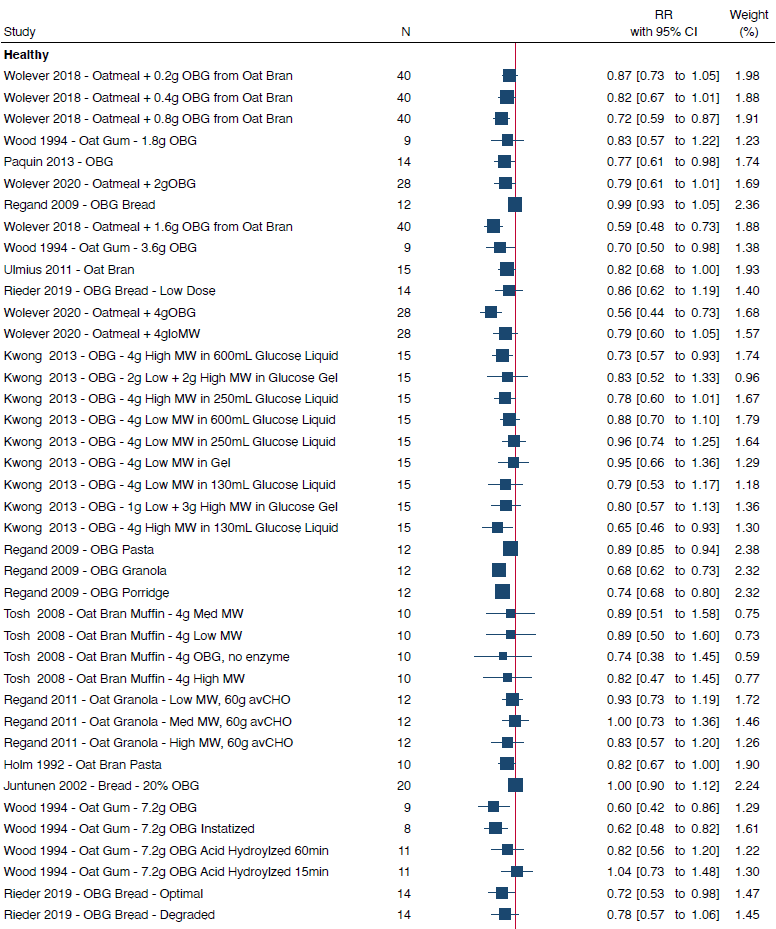


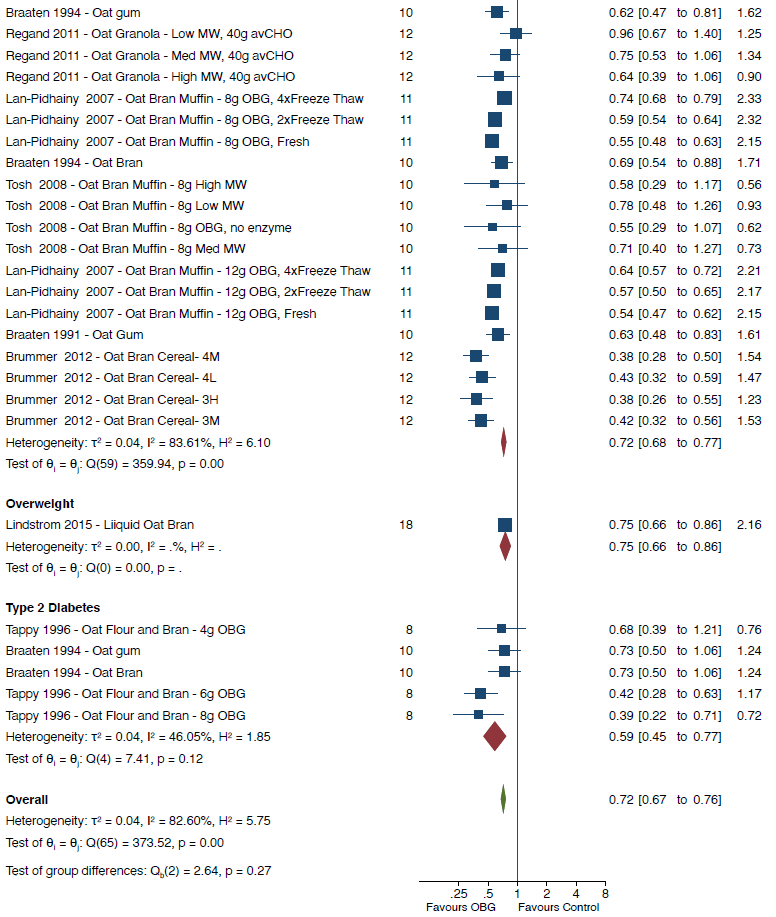
**Supplementary Figure 20** *(continued*

Data are expressed as ratio of means (RoMs) with 95% CIs using the generic inverse variance method modelled by random effects (DerSimonian-Laird). Trial comparisons within each subgroup are sorted from the lowest to the highest dose of oat β-glucan per 30g available carbohydrate portion The subgroup and total pooled effect estimates are represented by the red and green diamonds, respectively, with the size of the diamond representing the weight of the trial comparison in the overall analysis. Inter-study heterogeneity was assessed using the Cochran Q statistic and quantified using the I^2^ statistic, with P_Q_<0.10 and I^2^>50% considered to be evidence of substantial heterogeneity. Group differences were tested using subgroup meta-analysis where p<0.05 was considered significant.

# **Supplementary Figure 21**. RoB subgroup analysis of OBG on glucose iPeak


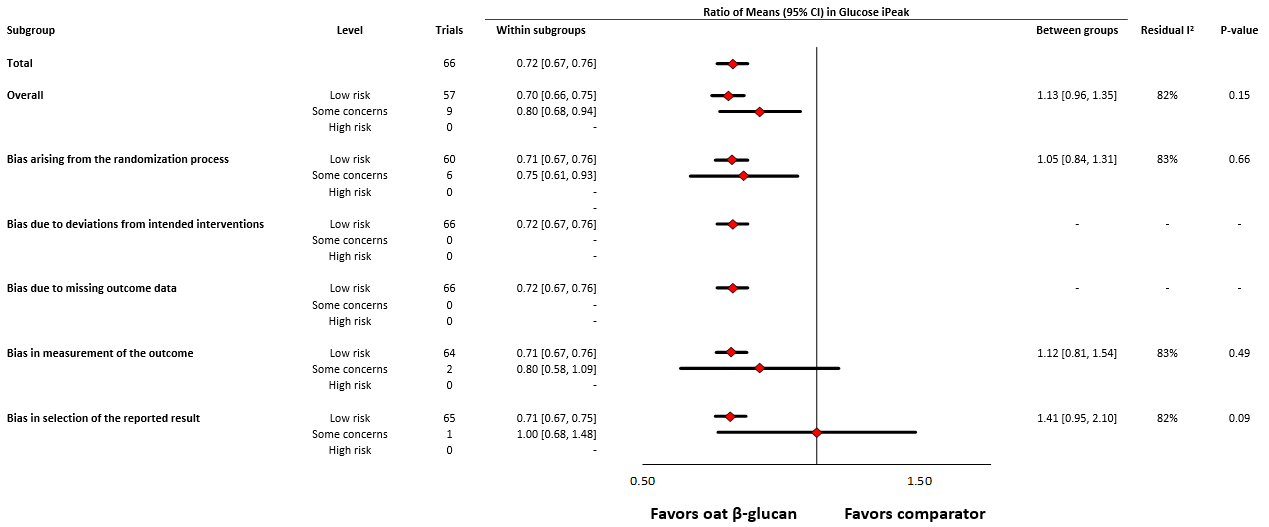


Data are expressed as ratio of means (RoM) with 95% confidence intervals (CIs). Differences between subgroups were tested using meta-regression and the significance level was reported as a p-value, where p<0.05 was considered significant. Pooled effect estimates for each subgroup are represented by the diamonds. The dashed line represents the pooled effect estimate for the overall analysis. The residual I^2^ value represents remaining between-study variation after adjustment for the subgroup.

# **Supplementary Figure 22.** Pooled effect estimates of OBG dose and insulin iAUC.


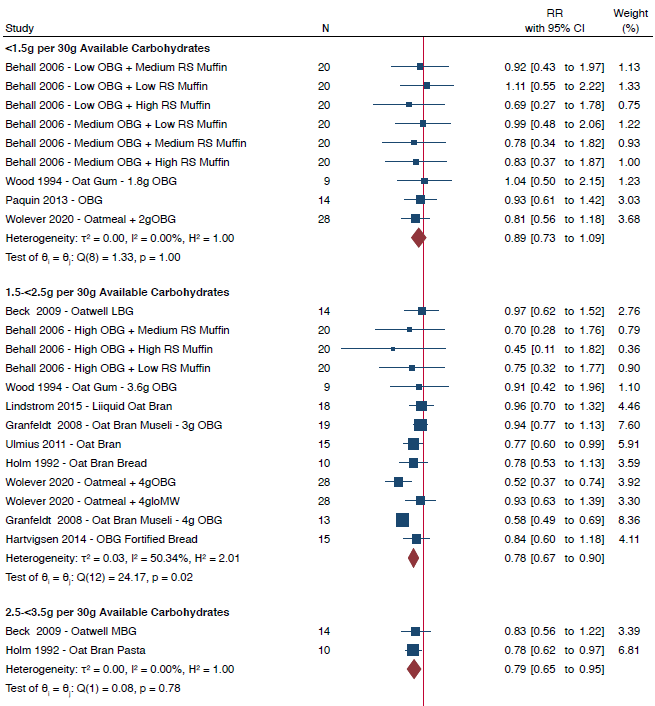


**Supplementary Figure 22** *(continued)*


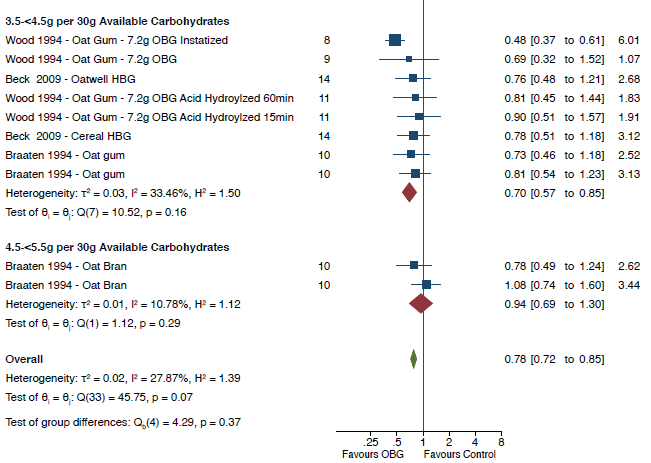


Data are expressed as ratio of means (RoMs) with 95% CIs using the generic inverse variance method modelled by random effects (DerSimonian-Laird). Trial comparisons within each subgroup are sorted from the lowest to the highest dose of oat β-glucan per 30g available carbohydrate portion The subgroup and total pooled effect estimates are represented by the red and green diamonds, respectively, with the size of the diamond representing the weight of the trial comparison in the overall analysis. Inter-study heterogeneity was assessed using the Cochran Q statistic and quantified using the I^2^ statistic, with P_Q_<0.10 and I^2^>50% considered to be evidence of substantial heterogeneity. Group differences were tested using subgroup meta-analysis where p<0.05 was considered significant.

# **Supplementary Figure 23.** Pooled effect estimates of OBG molecular weight and insulin iAUC.


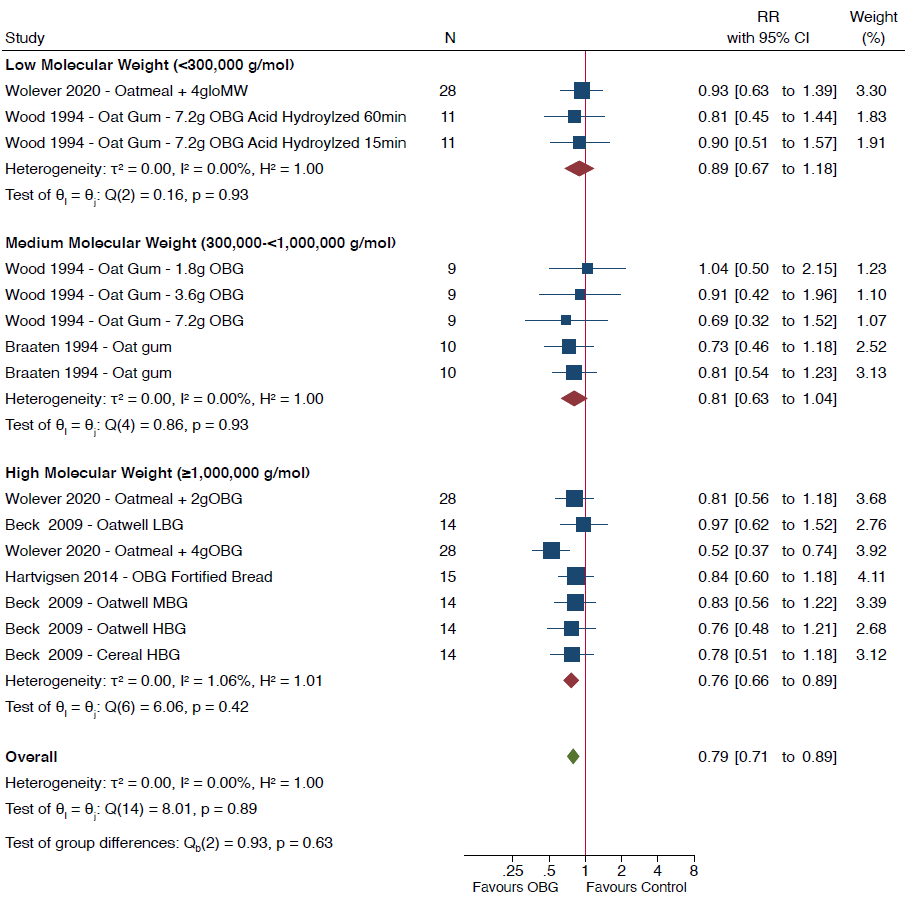
Molecular weight was reported in 12/31 trial comparisons. Data are expressed as ratio of means (RoMs) with 95% CIs using the generic inverse variance method modelled by random effects (DerSimonian-Laird). Trial comparisons within each subgroup are sorted from the lowest to the highest dose of oat β-glucan per 30g available carbohydrate portion The subgroup and total pooled effect estimates are represented by the red and green diamonds, respectively, with the size of the diamond representing the weight of the trial comparison in the overall analysis. Inter-study heterogeneity was assessed using the Cochran Q statistic and quantified using the I^2^ statistic, with P_Q_<0.10 and I^2^>50% considered to be evidence of substantial heterogeneity. Group differences were tested using subgroup meta-analysis where p<0.05 was considered significant.

# **Supplementary Figure 24.** Pooled effect estimates of OBG and insulin iAUC by type of comparator.


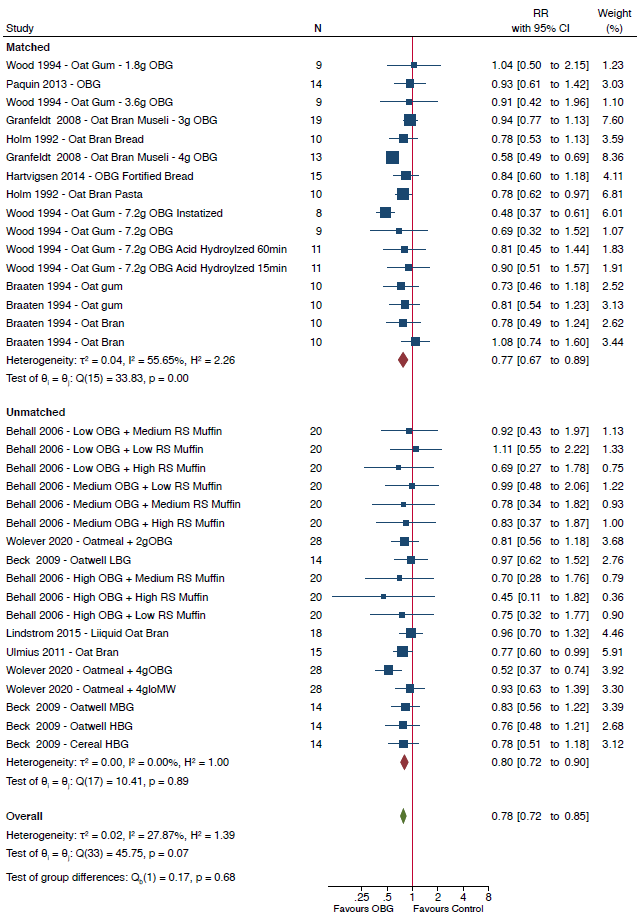


Data are expressed as ratio of means (RoMs) with 95% CIs using the generic inverse variance method modelled by random effects (DerSimonian-Laird). Trial comparisons within each subgroup are sorted from the lowest to the highest dose of oat β-glucan per 30g available carbohydrate portion The subgroup and total pooled effect estimates are represented by the red and green diamonds, respectively, with the size of the diamond representing the weight of the trial comparison in the overall analysis. Inter-study heterogeneity was assessed using the Cochran Q statistic and quantified using the I^2^ statistic, with P_Q_<0.10 and I^2^>50% considered to be evidence of substantial heterogeneity. Group differences were tested using subgroup meta-analysis where p<0.05 was considered significant.


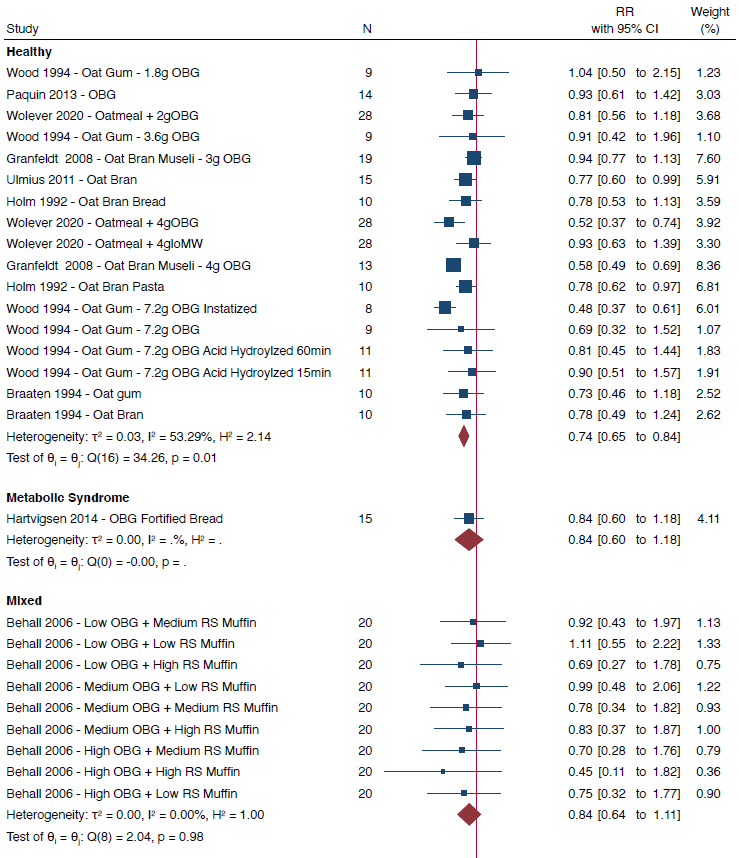
**Supplementary Figure 25.** Pooled effect estimates of OBG and insulin iAUC by health status.

**Supplementary Figure 25** *(continued)*


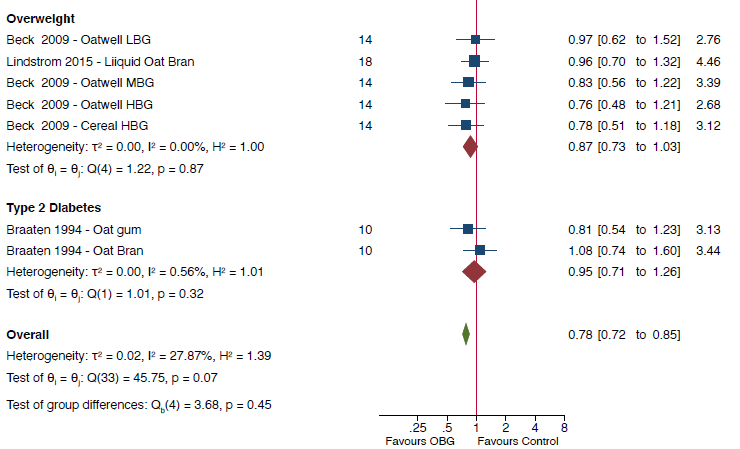


Data are expressed as ratio of means (RoMs) with 95% CIs using the generic inverse variance method modelled by random effects (DerSimonian-Laird). Trial comparisons within each subgroup are sorted from the lowest to the highest dose of oat β-glucan per 30g available carbohydrate portion The subgroup and total pooled effect estimates are represented by the red and green diamonds, respectively, with the size of the diamond representing the weight of the trial comparison in the overall analysis. Inter-study heterogeneity was assessed using the Cochran Q statistic and quantified using the I^2^ statistic, with P_Q_<0.10 and I^2^>50% considered to be evidence of substantial heterogeneity. Group differences were tested using subgroup meta-analysis where p<0.05 was considered significant.

**Supplementary Figure 26**. RoB subgroup analysis of OBG on insulin iAUC


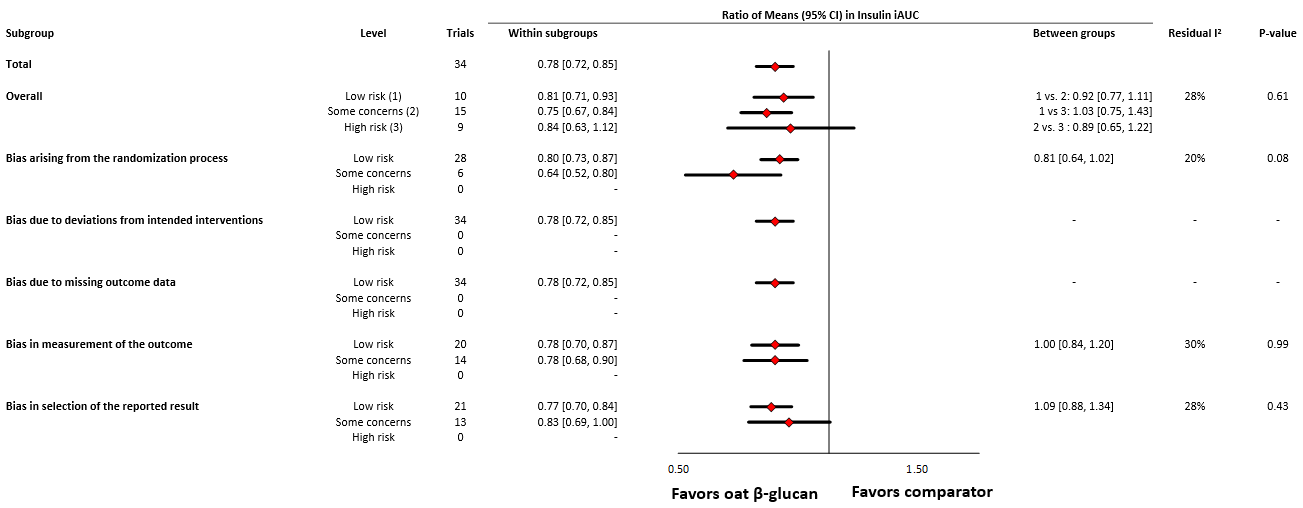


Data are expressed as ratio of means (RoM) with 95% confidence intervals (CIs). Differences between subgroups were tested using meta-regression and the significance level was reported as a p-value, where p<0.05 was considered significant. Pooled effect estimates for each subgroup are represented by the diamonds. The dashed line represents the pooled effect estimate for the overall analysis. The residual I^2^ value represents remaining between-study variation after adjustment for the subgroup.

# **Supplementary Figure 27.** Pooled effect estimates of OBG dose and insulin iPeak.


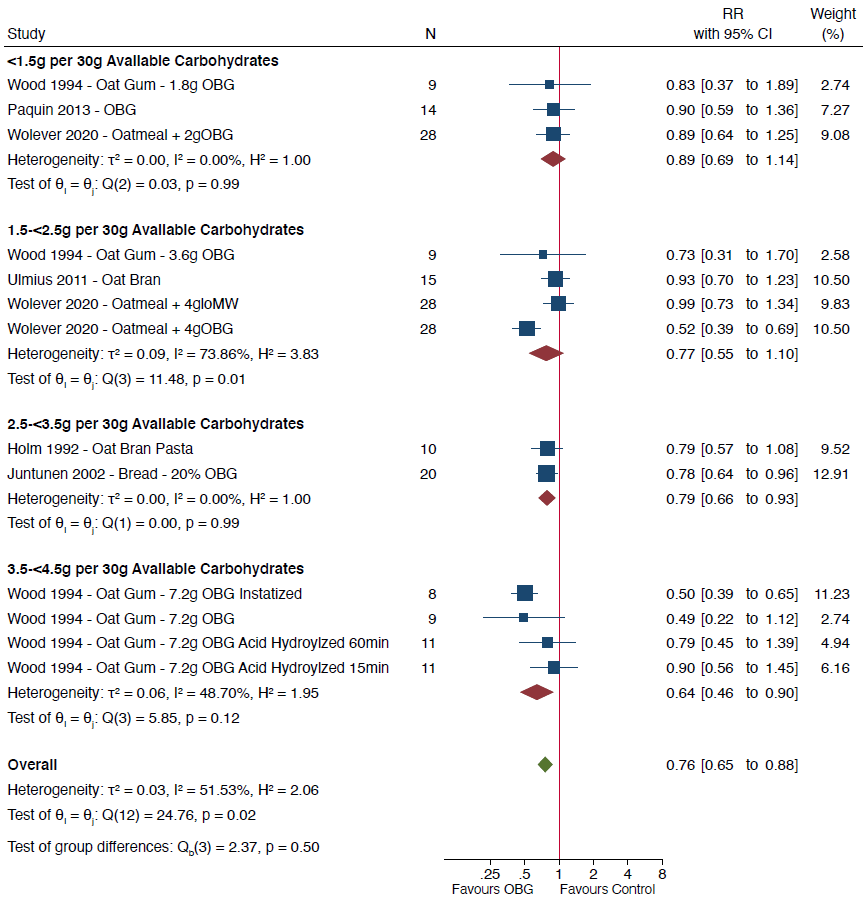


Data are expressed as ratio of means (RoMs) with 95% CIs using the generic inverse variance method modelled by random effects (DerSimonian-Laird). Trial comparisons within each subgroup are sorted from the lowest to the highest dose of oat β-glucan per 30g available carbohydrate portion The subgroup and total pooled effect estimates are represented by the red and green diamonds, respectively, with the size of the diamond representing the weight of the trial comparison in the overall analysis. Inter-study heterogeneity was assessed using the Cochran Q statistic and quantified using the I^2^ statistic, with P_Q_<0.10 and I^2^>50% considered to be evidence of substantial heterogeneity. Group differences were tested using subgroup meta-analysis where p<0.05 was considered significant.

# **Supplementary Figure 28.** Pooled effect estimates of OBG molecular weight and insulin iPeak.


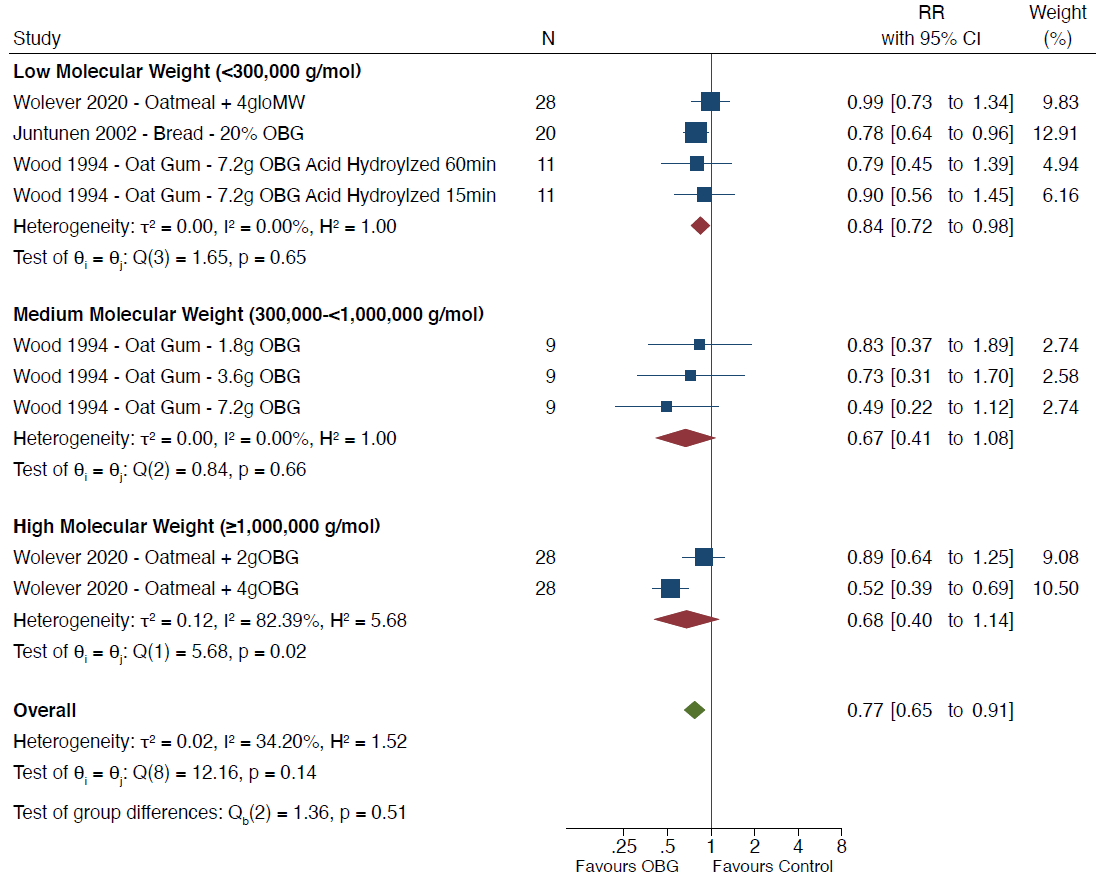


Molecular weight was reported in 6/10 trial comparisons. Data are expressed as ratio of means (RoMs) with 95% CIs using the generic inverse variance method modelled by random effects (DerSimonian-Laird). Trial comparisons within each subgroup are sorted from the lowest to the highest dose of oat β-glucan per 30g available carbohydrate portion The subgroup and total pooled effect estimates are represented by the red and green diamonds, respectively, with the size of the diamond representing the weight of the trial comparison in the overall analysis. Inter-study heterogeneity was assessed using the Cochran Q statistic and quantified using the I^2^ statistic, with P_Q_<0.10 and I^2^>50% considered to be evidence of substantial heterogeneity. Group differences were tested using subgroup meta-analysis where p<0.05 was considered significant.

#
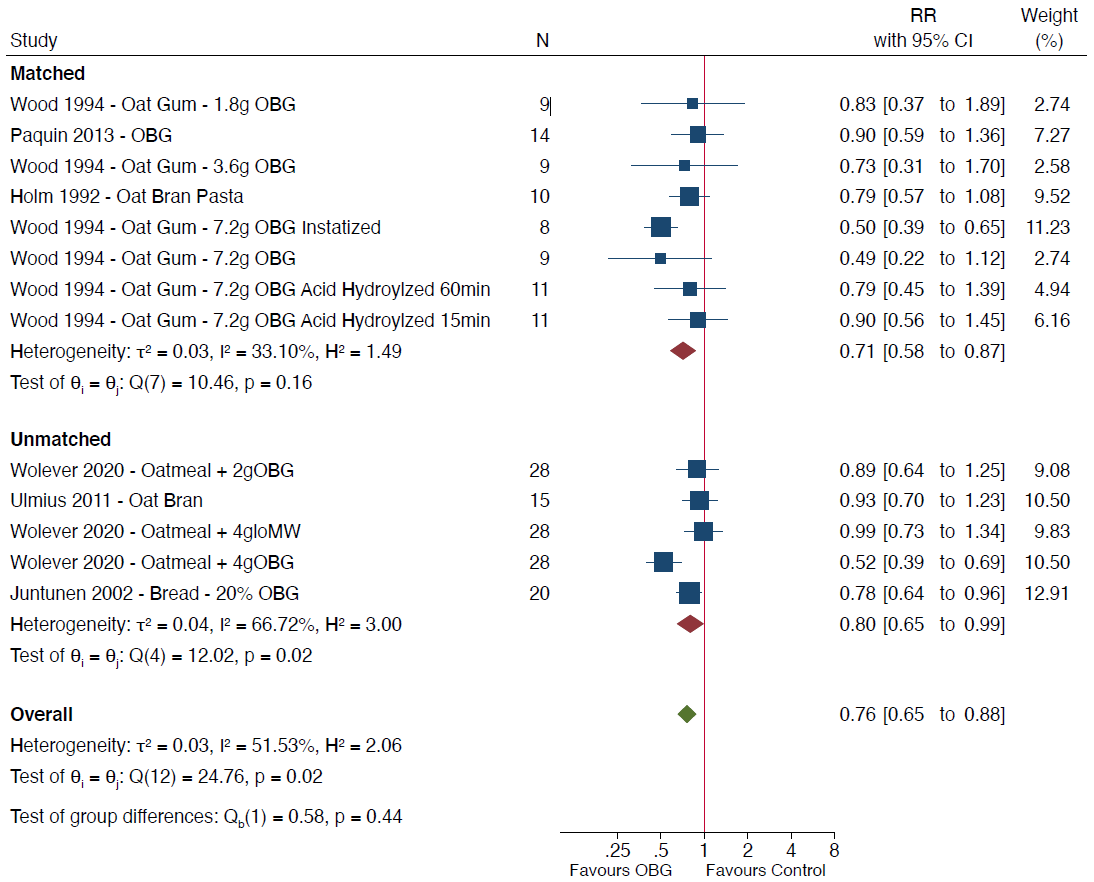
**Supplementary Figure 29.** Pooled effect estimates of OBG and insulin iPeak by type of comparator.

Data are expressed as ratio of means (RoMs) with 95% CIs using the generic inverse variance method modelled by random effects (DerSimonian-Laird). Trial comparisons within each subgroup are sorted from the lowest to the highest dose of oat β-glucan per 30g available carbohydrate portion The subgroup and total pooled effect estimates are represented by the red and green diamonds, respectively, with the size of the diamond representing the weight of the trial comparison in the overall analysis. Inter-study heterogeneity was assessed using the Cochran Q statistic and quantified using the I^2^ statistic, with P_Q_<0.10 and I^2^>50% considered to be evidence of substantial heterogeneity. Group differences were tested using subgroup meta-analysis where p<0.05 was considered significant.

# **Supplementary Figure 30**. RoB subgroup analysis of OBG on insulin iPeak


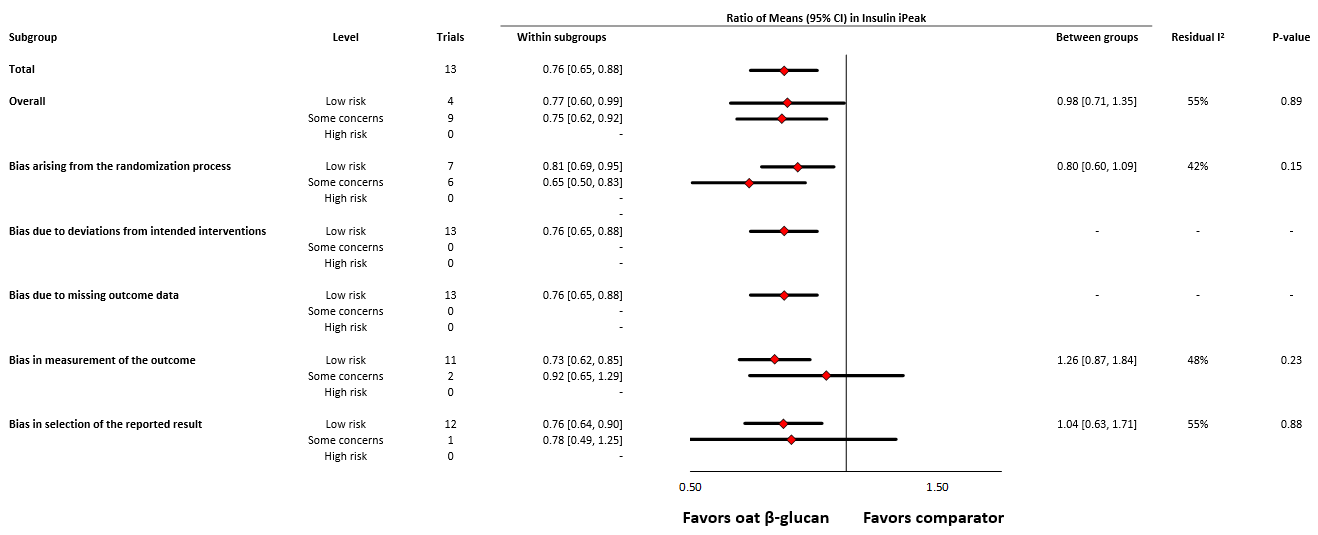


Data are expressed as ratio of means (RoM) with 95% confidence intervals (CIs). Differences between subgroups were tested using meta-regression and the significance level was reported as a p-value, where p<0.05 was considered significant. Pooled effect estimates for each subgroup are represented by the diamonds. The dashed line represents the pooled effect estimate for the overall analysis. The residual I^2^ value represents remaining between-study variation after adjustment for the subgroup.

# **Supplementary Figure 31.** Pooled effect estimates of OBG and insulin iPeak by health status.


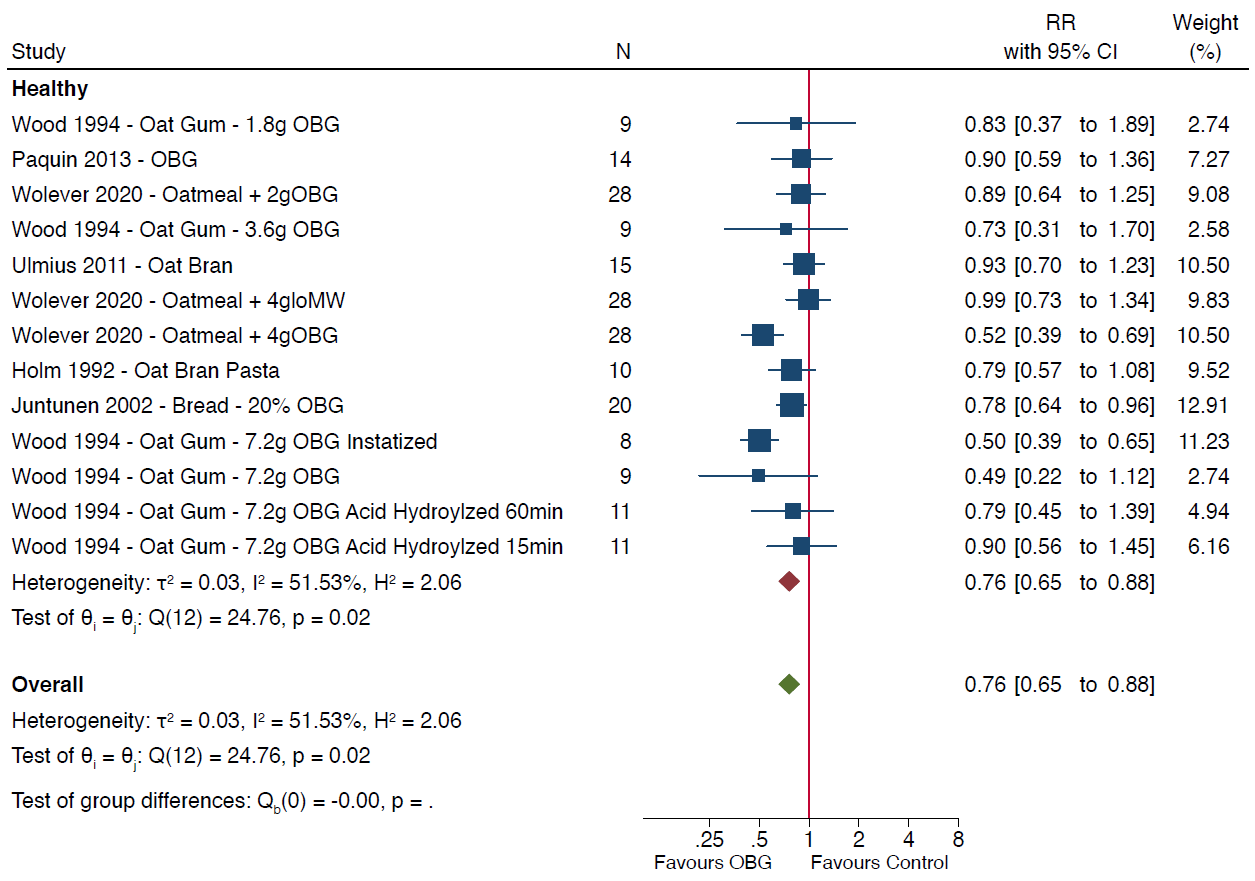


Data are expressed as ratio of means (RoMs) with 95% CIs using the generic inverse variance method modelled by random effects (DerSimonian-Laird). Trial comparisons within each subgroup are sorted from the lowest to the highest dose of oat β-glucan per 30g available carbohydrate portion The subgroup and total pooled effect estimates are represented by the red and green diamonds, respectively, with the size of the diamond representing the weight of the trial comparison in the overall analysis. Inter-study heterogeneity was assessed using the Cochran Q statistic and quantified using the I^2^ statistic, with P_Q_<0.10 and I^2^>50% considered to be evidence of substantial heterogeneity. Group differences were tested using subgroup meta-analysis where p<0.05 was considered significant.

# **Supplementary Figure 32.** Contour-enhanced funnel plot for the effect of OBG on the glucose and insulin iAUC and iPeak.

Funnel plot of natural logarithm ratio of means. The dots represent individual trial comparisons. The vertical solid red line represents the poled effect estimate and the dashed red lines represent the pseudo-95% confidence limits. The contour regions define the regions of statistically significant and nonsignificant levels with dark grey representing p>0.1, medium grey p >0.05 to ≤0.1, light grey p≤0.01 to p<0.05 and no shading p<0.01. The p-values were derived from quantitative assessment of publication bias by Egger’s and Begg’s tests set at a significance level of p<0.05.

**Supplementary Table 8.** GRADE certainty of the evidence assessment

| **No. of trial comparisons** | **Study design** | **GRADE ASSESSMENT** | | | | | | | | | | | | | | **No. of participants** | | | | **Effect**  **RoM (95% CI)** | **Certainty** |
| --- | --- | --- | --- | --- | --- | --- | --- | --- | --- | --- | --- | --- | --- | --- | --- | --- | --- | --- | --- | --- | --- |
|  |  | **Risk of bias** | | **Inconsistency** | | | **Indirectness** | | | **Imprecision** | | **Other considerations** | | | |  |  |  |  |  |  |
| **Glucose iAUC** | | | | | | | | | | | | | | | | | | | | | |
| 98 | Randomized and non-randomized trials | not serious | | | not serious^a^ | | | not serious | | not serious^b^ | | | | Dose-response^c^ | | | 508 | | | 0.77  (0.74 to 0.81) | **⨁⨁⨁⨁**  **HIGH** |
| **Glucose iPeak** | | | | | | | | | | | | | | | | | | | | | |
| 66 | Randomized and non-randomized trials | | not serious | | | serious^d^ | | | not serious | | not serious | | | Dose-response^c^ | | | 313 | | | 0.72  [0.64, 0.76] | **⨁⨁⨁⨁**  **HIGH** |
| **Insulin iAUC** | | | | | | | | | | | | | | | | | | | | | |
| 34 | Randomized and non-randomized trials | | not serious | | | not serious | | | not serious | | serious^e^ | | | Dose-response^c^ | | 231 | | | 0.78  [0.72, 0.85] | | **⨁⨁⨁⨁**  **HIGH** |
| **Insulin iPeak** | | | | | | | | | | | | | | | | | | | | | |
| 13 | Randomized and non-randomized trials | | not serious | | | not serious | | | not serious | | serious^e^ | | Dose-response^c^ | | 115 | | | 0.76  [0.65, 0.88] | | | **⨁⨁⨁⨁**  **HIGH** |

GRADE, Grading of Recommendations Assessment, Development, and Evaluation. iAUC, incremental area under the curve; iPeak, incremental peak rise; RoM, ratio of means.

^a^No downgrade as the presence of substantial heterogeneity (I^2^ =59.9%) was largely explained by dose (residual I^2^=7.2%, P_Q_=0.284).

^b^No downgrade for imprecision because while the 95% CI of the pooled effect estimate includes the clinical decision threshold for physiologically relevance (RoM=0.8), subgroup analyses indicated precise pooled effect estimates at doses >3.5g OBG/30g avCHO and from high molecular weight OBG.

^c^Dose response observed based on linear dose response analysis.

^d^Presence of significant heterogeneity (I^2^=82.6%) of which the source could not be identified through subgroup or sensitivity analyses.  ^e^95% CI includes the clinical decision threshold for physiologically relevance (RoM=0.8)
